# *Aspergillus Fumigatus* ZnfA, a Novel Zinc Finger Transcription Factor Involved in Calcium Metabolism and Caspofungin Tolerance

**DOI:** 10.3389/ffunb.2021.689900

**Published:** 2021-08-10

**Authors:** Clara Valero, Ana Cristina Colabardini, Patrícia Alves de Castro, Lilian Pereira Silva, Laure Nicolas Annick Ries, Lakhansing Pardeshi, Fang Wang, Marina Campos Rocha, Iran Malavazi, Roberto Nascimento Silva, Celso Martins, Patrícia Domingos, Cristina Pereira-Silva, Michael J. Bromley, Koon Ho Wong, Gustavo H. Goldman

**Affiliations:** ^1^Faculdade de Ciências Farmacêuticas de Ribeirão Preto, Universidade de São Paulo, Ribeirão Preto, Brazil; ^2^Medical Research Council Centre for Medical Mycology, University of Exeter, Exeter, United Kingdom; ^3^Faculty of Health Sciences, University of Macau, Macau, China; ^4^Genomics and Bioinformatics Core, Faculty of Health Sciences, University of Macau, Macau, China; ^5^Departamento de Genética e Evolução, Centro de Ciências Biológicas e da Saúde, Universidade Federal de São Carlos, São Paulo, Brazil; ^6^Faculdade de Medicina de Ribeirão Preto, Universidade de São Paulo, Ribeirão Preto, Brazil; ^7^Instituto de Tecnologia Química e Biológica António Xavier, Universidade Nova de Lisboa (ITQB NOVA), Oeiras, Portugal; ^8^Manchester Fungal Infection Group, Core Technology Facility, Faculty of Biology, Medicine and Health, Manchester Academic Health Science Centre, The University of Manchester, Manchester, United Kingdom; ^9^Manchester Academic Health Science Centre, Lydia Becker Institute of Immunology and Inflammation, Biology, Medicine and Health, The University of Manchester, Manchester, United Kingdom; ^10^Faculty of Health Sciences, Institute of Translational Medicine, University of Macau, Macau, China

**Keywords:** *Aspergillus fumigatus*, caspofungin, calcium, transcription factors, cell wall

## Abstract

Invasive pulmonary aspergillosis is a life-threatening fungal infection especially in the immunocompromised patients. The low diversity of available antifungal drugs coupled with the emergence of antifungal resistance has become a worldwide clinical concern. The echinocandin Caspofungin (CSP) is recommended as a second-line therapy but resistance and tolerance mechanisms have been reported. However, how the fungal cell articulates the response to CSP is not completely understood. This work provides a detailed characterization of ZnfA, a transcription factor (TF) identified in previous screening studies that is involved in the *A. fumigatus* responses to calcium and CSP. This TF plays an important role in the regulation of iron homeostasis and cell wall organization in response to high CSP concentrations as revealed by Chromatin Immunoprecipitation coupled to DNA sequencing (ChIP-seq) analysis. Furthermore, ZnfA acts collaboratively with the key TF CrzA in modulating the response to calcium as well as cell wall and osmotic stresses. This study therefore describes the existence of an additional, previously unknown TF that bridges calcium signaling and the CSP cellular response and further exposes the complex connections that exist among different pathways which govern stress sensing and signaling in *A. fumigatus*.

## Introduction

Invasive pulmonary aspergillosis is the most common human systemic infection caused by filamentous fungi (Brown et al., 2012; Bongomin et al., 2017). As an opportunistic disease, the immunocompromised population is particularly susceptible to acquire the infection (Krappmann, 2016). Despite the continuous increase of susceptible population to fungal infections, the number of available antifungal drugs is very limited. Azoles are usually used for aspergillosis treatment and prophylaxis but the emergence of azole resistance in the last years has become a matter of serious clinical concern (Chowdhary et al., 2017). In some centers, resistance levels are so high that azoles can no longer be used as sole first-line therapeutics (Verweij et al., 2015).

Echinocandins, such as caspofungin (CSP), are the newest class of antifungal drugs approved to treat invasive fungal infections. Their mode of action relies on inhibiting the 1,3-β-D-glucan synthase, which is responsible for the assembly of the β-D-glucan polymer, thus disrupting fungal cell wall integrity (Onishi et al., 2000). CSP is recommended as a second-line or salvage therapy for invasive pulmonary aspergillosis, even though it exhibits fungistatic activity against *A. fumigatus* (Walsh et al., 2008). Furthermore, the fungus has a remarkable ability to adapt to supra-inhibitory drug concentrations of CSP *via* a mechanism known as the caspofungin paradoxical effect (CPE), a phenomenon described first in *Candida* spp. (Hall et al., 1988). The CPE is linked to modifications in cell wall content and structure, such as a drastic increase in cell wall chitin and disappearance of cell wall β-1,3-glucan, as well as fungal morphology and growth (Steinbach et al., 2015; Wagener and Loiko, 2017). The calcium-calcineurin, Hsp90 and certain Mitogen-Activated Protein Kinase (MAPK) signaling pathways appear to play a major role in regulating these cellular and structural changes (Lamoth et al., 2014; Altwasser et al., 2015; Juvvadi et al., 2015; Ries et al., 2017).

Calcium is an essential secondary messenger and modulates the conformation of calcium-binding proteins, such as calmodulin, which activates calmodulin-dependent enzymes including the calcineurin phosphatase (Fox and Heitman, 2002; Cyert, 2003). In *A. fumigatus*, when cytosolic calcium increases in response to several stress stimuli, calcineurin dephosphorylates the key TF CrzA, which translocates to the nucleus and binds promoters of target genes (Soriani et al., 2008, 2010). The calcium-calcineurin pathway plays multiple important roles in *A. fumigatus* biology, including cell wall integrity, growth, antifungal resistance and virulence (Juvvadi et al., 2014). Additionally, we have reported that calcium signaling is also involved in compensatory cellular mechanisms such as the CPE by regulating the expression of specific chitin synthases (Ries et al., 2017) and cross-talk between different cell wall signaling pathways (de Castro et al., 2014), suggesting that calcium homeostasis in *A. fumigatus* might play a central role in various stress responses.

Recently, our group has performed two large phenotypic screenings of an *A. fumigatus* TF-null mutant library in the presence of high CaCl_2_ and CSP concentrations (500 mM and 16 μg/ml, respectively) (de Castro et al., 2019; Valero et al., 2020). As a result, Δ*znfA* (ΔAfu4g07090), a previously uncharacterized TF, was identified in both screenings. In this work we show that ZnfA plays a direct role in the regulation of siderophore biosynthesis and transport and cell wall organization in the presence of CSP. In addition, this work further unravels links among different signaling pathways that have been revealed as indicated by the collaboration between ZnfA and CrzA in the response to several stresses.

## Results

### The Zinc-Finger Tf ZnfA Is Involved in Calcium and CSP Tolerance Mechanisms

**Figure 1:**
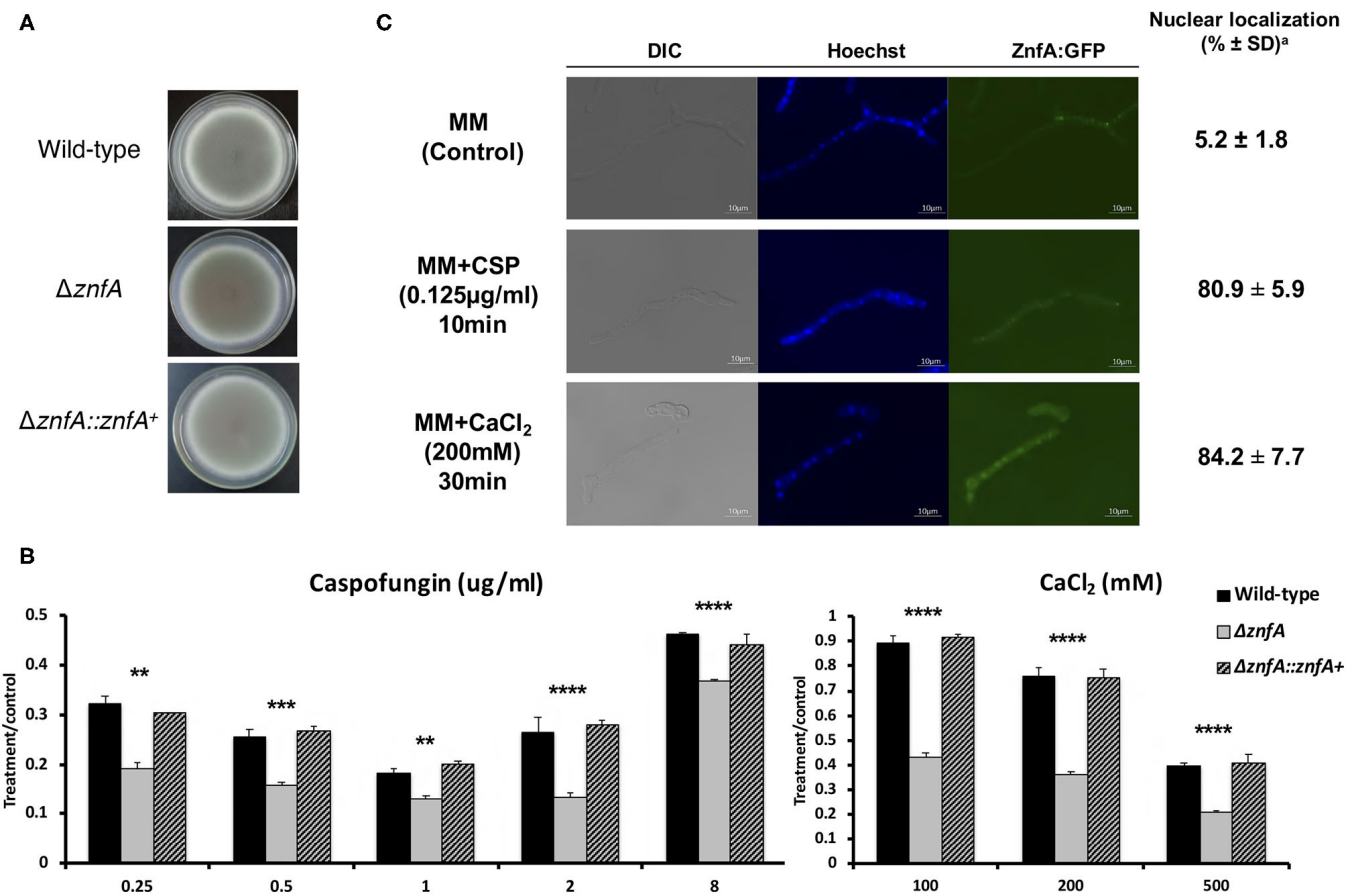
*A. fumigatus* ZnfA transcription factor is involved in calcium and caspofungin tolerance mechanisms. Growth phenotypes of the wild-type, Δ*znfA*, and Δ*znfA*::*znfA*^+^ strains grown for 5 days at 37°C in solid **(A)** MM and in the presence of increasing concentrations of **(B)** CaCl_2_ and Caspofungin. Standard deviations represent averages of results from three independent biological repetitions. Statistical analysis was performed using one-tailed, paired *t*-tests for comparisons to the control condition (^**^P < 0.01; ^***^P < 0.001; ^****^P < 0.0001). **(C)** ZnfA:GFP germlings were grown for 16 h at 37°C in MM and exposed to 0.125 μg/ml of Caspofungin for 10 min and 200 mM CaCl_2_ for 15 min for 30 min. Bar, 10 mm. ^*a*^The percentage of ZnfA:GFP nuclear localization is based on counting 300–500 nuclei in 50–100 hyphal germlings of biological triplicates.

The *znfA* gene (Afu4g07090) encodes a putative TF of 710 amino acids in length, with a molecular weight of 76.8 kDa and three zinc finger domains (C_2_H_2_ type), according to the SMART protein domain annotation software (http://smart.embl-heidelberg.de/). As observed in Figure 1A, the Δ*znfA* mutant did not show reduced growth in solid minimal medium (MM), indicating that deletion of *znfA* gene does not affect the vegetative growth of the fungus in the presence of standard, non-stress laboratory conditions. Furthermore, as we previously reported, Δ*znfA* was more sensitive to CaCl_2_ and CSP (Figure 1B), and had a reduced CPE in comparison to the wild-type and complemented strains (de Castro et al., 2019; Valero et al., 2020). Additionally, the deletion strain exhibited reduced growth at 30°C (Supplementary Figure 1A) and slightly increased growth in the presence of the calcium chelating agent EGTA at low concentrations (Supplementary Figure 1B). Re-introduction of *A. fumigatus znfA* gene in the Δ*znfA* background strain restored growth when the fungus was exposed to the aforementioned stressors. These results emphasize that ZnfA is involved in the response to calcium and CSP stresses.

To investigate ZnfA cellular localization, we constructed a strain that produces a functional ZnfA:GFP fusion protein (Supplementary Figure 2A). As showed in Figure 1C, ZnfA:GFP localized within the cytosol in the absence of any stress, but was translocated to the nucleus when 200 mM CaCl_2_ (~84%) or 0.125 μg/ml of CSP (~81%) were added to the culture medium. Nuclear localization of ZnfA:GFP increased over time (10–30 min, 65–84%) after the addition of CaCl_2_, but remained constant after CSP treatment.

### ZnfA Remains Phosphorylated After Calcium Treatment

**Figure 2:**
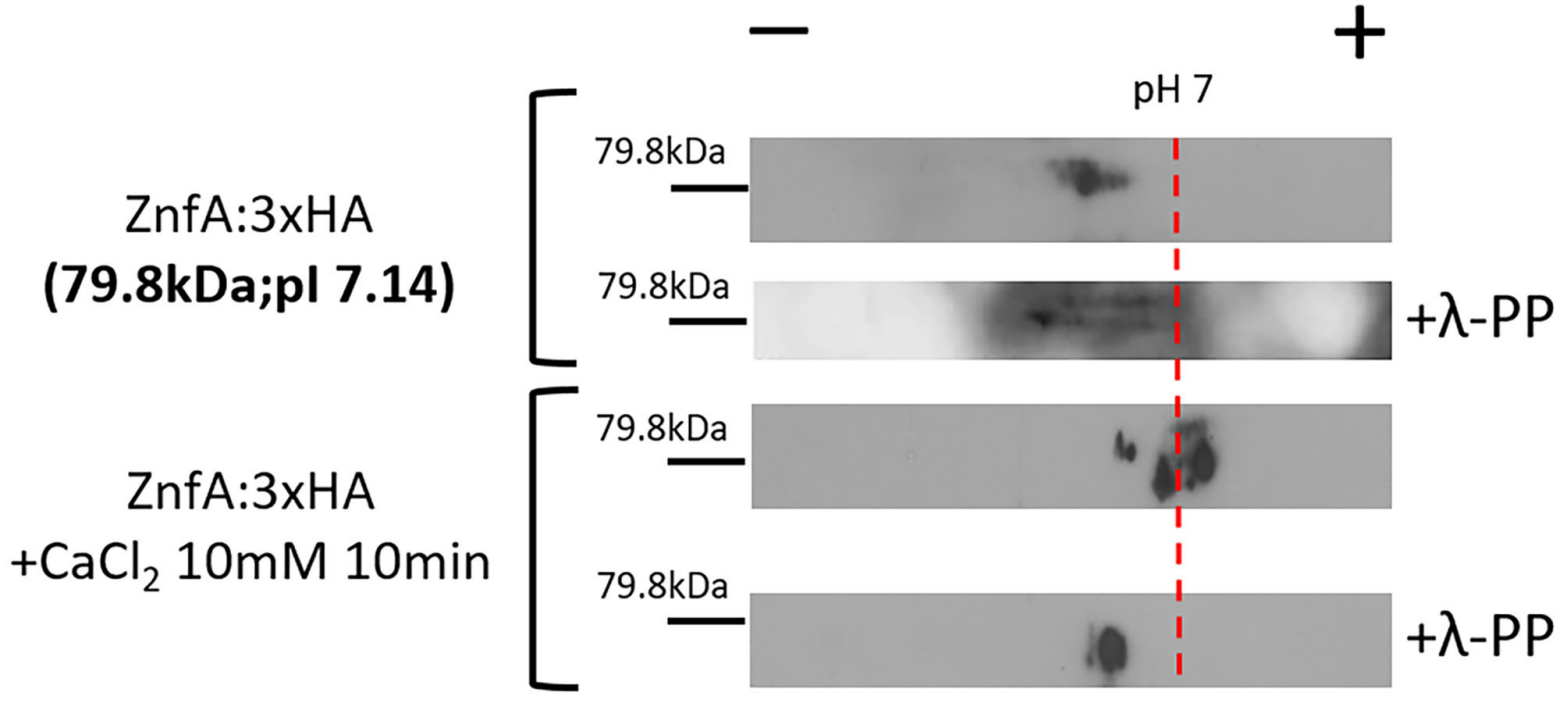
*A. fumigatus* ZnfA:3xHA is not dephosphorylated upon calcium stress. ZnfA:3xHA strain was grown for 24 h at 37°C and treated or not with CaCl_2_ 10 mM for 10 min. Protein extracts were subjected to immunoprecipitation and left untreated or treated with lambda phosphatase (λ-PP). Bidimensional gel electrophoresis coupled with western blotting was then carried out by using anti-HA antibody against the HA epitope to detect ZnfA:3xHA. pI, isoelectric point.

Both CrzA and ZipD calcium-responsive TFs are dephosphorylated upon calcium stress before translocating to the nucleus (de Castro et al., 2019; Shwab et al., 2019). To investigate if ZnfA is under phosphoregulation, we constructed a strain that expresses the ZnfA protein C-terminally fused to a 3xHA-tag epitope and has no detectable difference in growth under CaCl_2_ or CSP conditions comparing to the wild-type strain (Supplementary Figure 2A), which was subjected to bidimensional electrophoresis analysis. Protein extracts were prepared from mycelia of the ZnfA:3xHA strain that were untreated or treated with CaCl_2_ 10 mM for 10 min. Then, proteins were immunoprecipitated using anti-HA antibodies coupled to magnetic beads and one replicate for ZnfA:3xHA strain for each condition was treated with a lambda protein phosphatase (λ-PP) for 1 h at 30°C, which was used as a positive control for dephosphorylation. Bidimensional electrophoresis coupled to immunoblotting analysis showed that after calcium exposure, ZnfA had increased phosphorylation since there is an increase on acidic forms of the protein (positive pH), in contrast to λ-PP treatment condition (Figure 2). This result indicates that ZnfA is still phosphorylated upon calcium exposure.

### ZnfA Binds to the Promoter Regions of Genes Involved in Siderophore Biosynthesis and Transport and Cell Wall Remodeling

**Figure 3:**
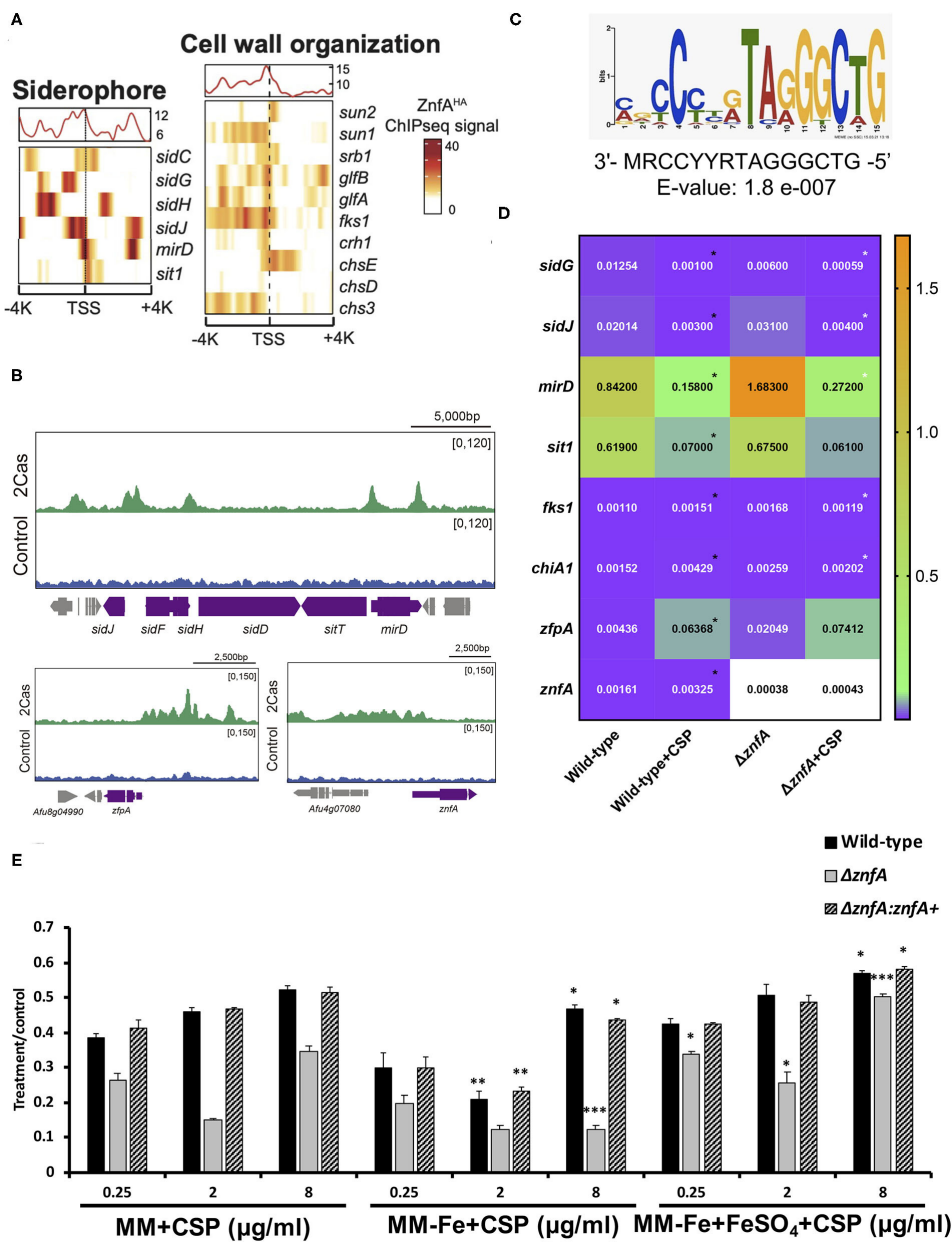
ZnfA binds to the promoter regions of specific genes encoding for siderophore biosynthesis and transport and cell wall remodeling after caspofungin exposure. Chromatin immunoprecipitation coupled to DNA sequencing (ChIP-seq) of the ZnfA:3xHA strain when grown for 16 h at 37°C and treated or not with 2 μg/ml of CSP for 1 h was assessed. **(A)** Heat map depicting ChIP-seq signal for genes involved in siderophore biosynthesis and transport and cell wall organization. **(B)** Selected examples of peaks identified in the promoter region of genes belonging to the siderophore biosynthetic cluster, *zfpA* and *znfA* genes in control and CSP (2cas) conditions. **(C)** DNA sequence and E-value of the putative ZnfA binding motif identified by MEME-ChIP analysis, that was enriched in the presence of CSP. **(D)** Heatmap showing normalized expression ratios of selected genes whose promoter regions were bound by ZnfA in the presence of CSP (2 μg/ml) according to the ChIP-seq analysis. The heatmap graph was created by using GraphPad Prism 8. Statistical analysis was performed using one-tailed, paired *t*-tests for comparisons between the control and caspofungin condition for the wild-type strain (Wild-type vs. Wild-type+CSP; black asterisks) and both strains in the presence of the drug (Wild-type+CSP vs. Δ*znfA*+CSP; white asterisks) **(E)** Radial growth phenotypes were determined for the wild-type, Δ*znfA* and Δ*znfA::znfA*^+^ strains after 5 days of growth at 37°C in MM, MM-Fe, and MM-Fe supplemented with FeSO_4_ 200 μM with and without CSP. Standard deviations represent averages of results from three independent biological repetitions. Statistical analysis was performed using one-tailed, paired *t*-tests for comparisons to the control condition (MM plus CSP) (^*^P < 0.05; ^**^P < 0.01).

To investigate which genes are directly regulated by ZnfA in the presence of CSP, genome-wide binding sites were determined by ChIP-seq using the ZnfA:3xHA strain grown for 16 h in MM with or without CSP (2 μg/ml) treatment for 1 h. Multiple ZnfA binding sites were identified throughout the genome (*n* = 93 in control and 752 in the CSP condition), of which 31 were exclusively bound in the control, 30 in both control and CSP, and 747 exclusively upon CSP treatment (Supplementary Table 1). The strongest ZnfA bindings sites (i.e., higher fold increase values) were observed in the CSP-exclusive condition with enrichment of genes that encode proteins involved in siderophore biosynthesis and transport and cell wall modification, such as genes related to chitin biosynthesis and degradation, β-1,3-glucan synthesis and modification and carbohydrate mobilization (Figure 3A; Table 1). We also identified ZnfA binding to the promoter regions of genes encoding calmodulin (Afu4g10050), annexin (Afu2g13890), and calcium/calmodulin-dependent protein kinase (Afu2g13680) under the CSP exposure condition (Supplementary Table 1). These observations suggest that ZnfA directly regulates genes involved in cell wall organization and iron homeostasis in the presence of CSP. Remarkably, ZnfA was found to bind its own promoter region after CSP treatment (almost 2-fold increase in comparison to the control condition), albeit the binding is not as typical as a point source peak, indicating that ZnfA could autoregulate its induction in the presence of the drug (Figure 3B).

**Table 1:** List of the main ZnfA:3xHA target genes identified in the ChIP-seq analysis enriched in the caspofungin exclusive condition.

**Upstream closest gene**	**Fold enrichment**	**Gene name**	**Description**	**Categorization**
Afu1g17270	2.07561	*fre2*	Metalloreductase involved in response to iron starvation; repressed by iron through SreA-dependent regulatory system	Iron homeostasis
Afu1g17200	2.17129	*sidC*	Putative non-ribosomal peptide synthetase (NRPS) involved in ferricrocin siderophore biosynthesis; expression is up-regulated by iron starvation and downregulated under iron-replete conditions	Iron homeostasis
Afu2g05830	2.30769	*acuK*	Putative transcription factor involved in regulation of gluconeogenesis and acquisition of iron; required for virulence	Iron homeostasis
Afu7g06060	2.90909	*sit1*	Putative siderophore transporter; SrbA-regulated during hypoxia	Iron homeostasis
Afu3g03410	2.73843	*sidH*	Enoyl-CoA hydratase/isomerase family protein; mevalonyl-CoA hydratase; PTS1 receptor-mediated peroxisomal localization is essential for triacetylfusarinine C (TAFC) biosynthesis	Iron homeostasis
Afu6g12870	3.04948	*atm1*	ABC iron exporter; transcript up-regulated in conidia exposed to neutrophils	Iron homeostasis
Afu3g03390	2.90033	*sidJ*	Predicted siderophore biosynthesis lipase/esterase involved in siderophore hydrolysis	Iron homeostasis
Afu3g03650	3.48584	*sidG*	Putative acetyltransferase with a predicted role in iron metabolism; located in an iron-regulated gene cluster; fusarinine C acetyltransferase; SrbA-regulated during hypoxia	Iron homeostasis
Afu3g03440	4.33437	*mirD*	Putative siderophore transporter; expression upregulated under low iron conditions; SrbA-regulated during hypoxia	Iron homeostasis
Afu7g05450	1.92998	*sun1*	Novel beta-1,3-glucan modifying enzyme involved in fungal morphogenesis	Cell wall organization
Afu8g05620	1.98339	*chs3*	Ortholog(s) have enzyme activator activity and role in cellular protein localization, division septum assembly, fungal-type cell wall chitin biosynthetic process, regulation of fungal-type cell wall beta-glucan biosynthetic process	Cell wall organization
Afu2g03120	2.13816	*utr2*	Putative cell wall glucanase; transcript induced by exposure to human airway epithelial cells	Cell wall organization
Afu4g11510	2.27008	*srb1*	GDP-mannose pyrophosphorylase, which catalyses the synthesis of GDP-mannose from GTP and mannose-1-phosphate in cell wall biosynthesis	Cell wall organization
Afu1g15440	2.27273	*ags3*	Putative alpha(1–3) glucan synthase; mutants show increased lung invasion and fungal burden in immunosuppressed mice, increased rate of germination, increased melanin production	Cell wall organization
Afu3g12700	2.20791	*glfB*	Putative UDP-galactofuranose transporter	Cell wall organization
Afu6g08510	2.47286	*crh1*	Putative cell wall biosynthesis protein	Cell wall organization
Afu1g12600	2.51309	*chsD*	Putative chitin synthase-like gene with a predicted role in chitin biosynthesis	Cell wall organization
Afu3g12690	2.42537	*glfA*	Putative UDP-galactopyranose mutase, enzyme in the first step of galactofuranose biosynthesis; mutant unmasks mannan residues on the cell surface, which is thought to contribute to increased cell adhesion	Cell wall organization
Afu2g13440	2.59481	*chsE*	Putative class V chitin synthase; required for normal hyphal growth, conidiation and normal conidiophore development; mutants have decreased chitin content	Cell wall organization
Afu1g13940	2.83871	*sun2*	Predicted adhesin-like protein; novel beta-1,3-glucan modifying enzyme involved in fungal morphogenesis	Cell wall organization
Afu6g12400	2.48062	*fks1*	Putative 1,3-beta-glucan synthase catalytic subunit, major subunit of glucan synthase; predicted transmembrane protein; essential	Cell wall organization
Afu5g03760	2.85412	*chiA1*	Putative class III chitinase; predicted GPI-anchoring sequence; protein and transcript repressed by caspofungin treatment	Cell wall organization
Afu8g05710	3.45946	*mfsA*	Putative major facilitator superfamily (MFS) sugar transporter; calcium induced; transcript up-regulated in conidia exposed to neutrophils	Cell wall organization
Afu6g04270	3.71046	Afu6g04270	Putative MFS sugar transporter; transcript up-regulated in conidia exposed to neutrophils	Cell wall organization
Afu6g04270	3.71046	Afu6g04270	Putative MFS sugar transporter; transcript up-regulated in conidia exposed to neutrophils	Cell wall organization

As a means to identify DNA binding motifs enriched in the presence of CSP, a MEME (Multiple Expectation maximizations for Motif Elicitation)-ChIP analysis was carried out on the 500 bp regions surrounding the peak summit identified in the ChIP-seq analysis. Interestingly, only one reliable motif was found (3′- MRCCYYRTAGGGCTG-5′; E-value: 1.8 e-007) (Figure 3C).

With the purpose to better understand the role of ZnfA in the regulation of the direct target genes identified in the ChIP-seq analysis, the gene expression of a set of 8 of those genes was determined by RT-qPCR following the same experimental design used to performed the ChIP-seq analysis (Figure 3D). Four of the selected genes encode proteins involved in iron homeostasis: (i) *mirD* (Afu3g03440) and (ii) *sit1* (Afu7g06060); that have been identified as siderophore transporters (Moloney et al., 2016; Park et al., 2016) and (iii) *sidG* (Afu3g03650) and (iv) *sidJ* (Afu3g03390); both of them involved in siderophore biosynthesis (Schrettl et al., 2007; Gründlinger et al., 2013). Two other genes encode two proteins involved in cell wall remodeling: *fks1* (Afu6g12400), which encodes the 1,3-β-D-glucan synthase that is in turn the target of CSP (Beauvais et al., 2001) and *chiA1* (Afu5g03760), a chitinase that has the potential to hydrolyze chitin, one of the main structural components of the fungal cell wall (Taib et al., 2005). We also included in the set, two TFs: *zfpA* (Afu8g05010) and *znfA* (Afu4g07090) itself. The highest modulation was observed in the siderophore transporters *mirD* and *sit1*, that were down-regulated in the presence of CSP in the wild-type strain. Only *mirD* was significantly up-regulated in the Δ*znfA* mutant upon CSP treatment when comparing to the wild-type strain in the same condition. *sidG* and *sidJ* were also down-regulated in the wild-type strain treated with CSP. In the Δ*znfA* mutant upon CSP treatment, *sidG* replicated the down-regulated result while *sidJ* was up-regulated. In contrast, cell wall remodeling (*fks1* and *chiA1*) and TF (*zfpA* and *znfA*) genes were up-regulated in the wild-type strain upon treatment with the drug. Only *fks1* and *chiA1* were down-regulated in the Δ*znfA* background strain in the presence of CSP. Taken together, these results suggest that *znfA* is a repressor of the synthesis and transport of siderophores in the presence of CSP, while enhances the expression of Fks1, ChiA1, ZfpA and itself.

Since the ChIP-seq analysis revealed a role of ZnfA in iron homeostasis in the presence of CSP, radial growth experiments of the wild-type, Δ*znfA* and Δ*znfA::znfA*^+^ strains in different conditions of iron availability and in combination with CSP were determined (Figure 3E). The Δ*znfA* mutant lost the CPE completely when grown in iron-deficient MM (MM-Fe) in comparison to standard MM, while it was conserved in the wild-type and complemented strains despite a slight reduction in absolute growth. Of note, the CPE was restored and even potentiated in the Δ*znfA* mutant strain upon iron supplementation of MM-Fe media. These results suggest that iron availability is important for CPE in the Δ*znfA* mutant.

### Genetic Interactions Between ZnfA and the TFs CrzA and ZipD

**Figure 4:**
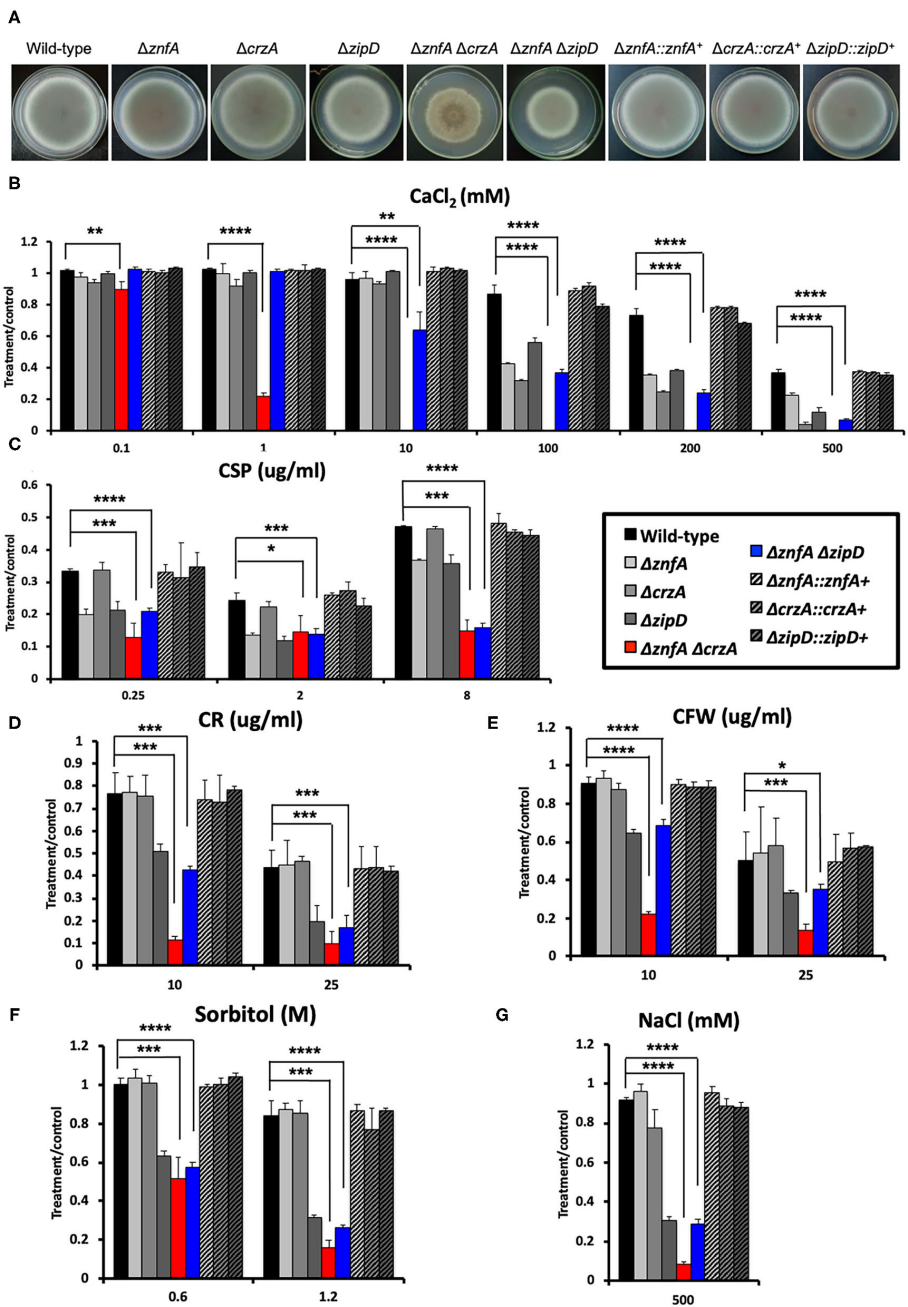
*A. fumigatus* ZnfA, CrzA and ZipD transcriptions factors collaborate in the response to calcium and caspofungin stresses. **(A)** Growth phenotypes of the wild-type, Δ*znfA*, Δ*crzA*, Δ*zipD*, Δ*znfA* Δ*crzA*, Δ*znfA* Δ*zipD*, and corresponding complemented strains grown for 5 days at 37°C in solid MM. Radial growth determination of aforementioned strains in the presence of increasing concentrations of **(B)** calcium chloride (CaCl_2_), (**C)** caspofungin (CSP), **(D)** Congo red (CR), **(E)** calcofluor white (CFW), **(F)** sorbitol and **(G)** sodium chloride (NaCl). Standard deviations represent three biological replicates and statistical analysis was performed using one-tailed, paired *t*-test (^*^P < 0.05; ^**^P < 0.01; ***P < 0.001; ^****^P < 0.0001).

To investigate possible interactions between ZnfA and other TFs involved in calcium signaling and CSP tolerance, two double null mutants, Δ*znfA* Δ*crzA* and Δ*znfA*Δ*zipD*, were constructed (Supplementary Figure 2B). Both CrzA and ZipD were identified to be involved in calcium and CSP stress responses (Ries et al., 2017; de Castro et al., 2019). As observed in Figure 4A, both Δ*znfA*Δ*crzA* and Δ*znfA*Δ*zipD* showed a decrease in radial growth in solid MM when compared to the wild-type and single deletion mutant strains (25% of reduction, approximately). Furthermore, Δ*znfA* Δ*crzA* exhibited strong defects in conidiation (Supplementary Figure 2C). Radial growth experiments in solid MM supplemented with different stressors revealed that Δ*znfA* Δ*crzA* was extremely sensitive to CaCl_2_, cell wall damaging agents (Congo red and calcofluor white) and high osmotic concentrations (sorbitol and NaCl), while the CPE was completely lost (Figures 4B–G). Additionally, the Δ*znfA* Δ*crzA* strain had significantly reduced growth at 30°C than the wild-type and Δ*znfA*, whilst growing better in the presence of EGTA (Supplementary Figures 1C,D). In contrast, Δ*znfA* Δ*zipD* behaved similarly to Δ*zipD* in all here tested conditions, except for being more sensitive to high CaCl_2_ concentrations and, just like the Δ*znfA* Δ*crzA* strain, it entirely lost the CPE (Figures 4B–G; Supplementary Figure 1). These results suggest that ZnfA, CrzA and ZipD act additively in the calcium and CSP cell response, while ZnfA and CrzA but not ZipD cooperate in responding to cell wall and osmotic stresses.

### ZnfA, CrzA and ZipD Transcription Factors Are Involved in Cell Wall Organization

**Figure 5:**
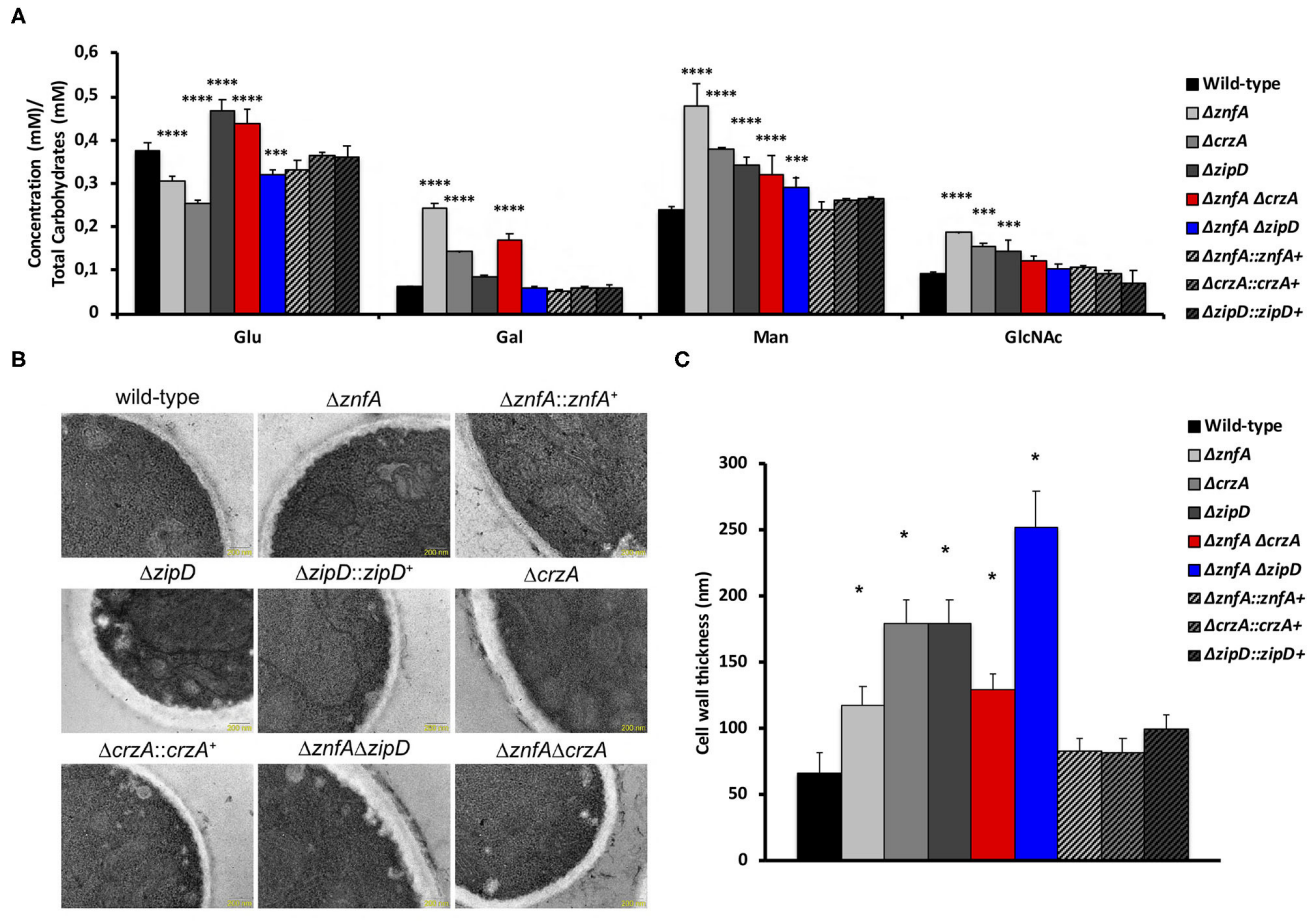
*A. fumigatus* ZnfA, CrzA and ZipD transcription factors are required for the correct organization of the cell wall. **(A)** Cell wall sugar content as determined by high performance liquid chromatography (HPLC), in mycelial extracts of *A. fumigatus* strains included in this work when grown for 16 h in MM at 37°C. Standard deviations represent three biological replicates and statistical analysis was performed using Two-way ANOVA test followed by Dunnett's multiple comparison test (^***^P < 0.001; ^****^P < 0.0001). Glu, Glucose; Gal, Galactose; Man, Mannose; GlcNAc; N-acetyl Glucosamine. **(B)** TEM images of mycelial sections of wild-type, Δ*znfA*, Δ*znfA::znfA*^+^, Δ*zipD*, Δ*zipD::zipD*^+^, Δ*crzA*, Δ*crzA::crzA*^+^, Δ*znfA* Δ*zipD* and Δ*znfA* Δ*crzA* strains mycelia grow in MM at 37°C. Magnification bars: 200 nm. **(C)** The cell wall thickness of 100 sections from different germlings was measured and plotted on the graph. Average ± SD are shown. Statistics were determined by one-way ANOVA with a Dunnett's multiple comparison test. Asterisks indicate significant differences between each mutant strain compared to the wild-type strain (^*^p < 0.0001).

In order to investigate the importance of ZnfA, CrzA, and ZipD for cell wall composition, high-performance liquid chromatography (HPLC) analysis was performed to quantify total cell wall polysaccharides. Quantities were normalized by the total cell wall carbohydrate content. All mutant strains displayed higher quantities of mannose (Man) than the wild-type and complemented strains, but only the Δ*znfA*, Δ*crzA* and Δ*znfA* Δ*crzA* strains exhibited significantly higher levels of galactose (Gal) in the cell wall. The Δ*znfA*, Δ*crzA* and Δ*znfA* Δ*zipD* strains contained lower glucose (Glu) concentrations than the wild-type and complemented strains, while the Δ*zipD* and Δ*znfA* Δ*crzA* strains displayed higher levels of this polysaccharide in the cell wall. Finally, just the single deletion mutants had higher amounts of N-acetyl glucosamine (GlcNAc) in their cell walls (Figure 5A). These results suggest that ZnfA, CrzA and ZipD are required for regulating the maintenance of sugar concentrations in the fungal cell wall.

Transmission electron microscopy (TEM) showed the cell wall of all the single and double mutants was thicker than the wild-type and complemented strain (Figures 5B,C). The Δ*znfA* Δ*zipD* mutant exhibited the thickest cell wall, which may be due to the synergistic effect of the absence of the two TFs, however this increase did not correlate with any particular increase of any cell wall polysaccharide. In contrast, such effect was not observed in the Δ*znfA* Δ*crzA* mutant strain, which indicates that some compensatory mechanism may be engaged as a result of the deletion of *znfA* and *crzA* genes. Therefore, ZnfA, CrzA and ZipD also influence fungal cell wall architecture.

Although several changes in cell wall composition and structure of deletion mutant strains have been identified, no differences in antifungal susceptibility patterns were detected against azoles and amphotericin B when compared with the wild-type and complemented strains (Supplementary Table 2).

### The *A. fumigatus* Δ*znfA* Strain Is Still Virulent in an Immunodeficient Mouse Model of Invasive Pulmonary Aspergillosis

**Figure 6:**
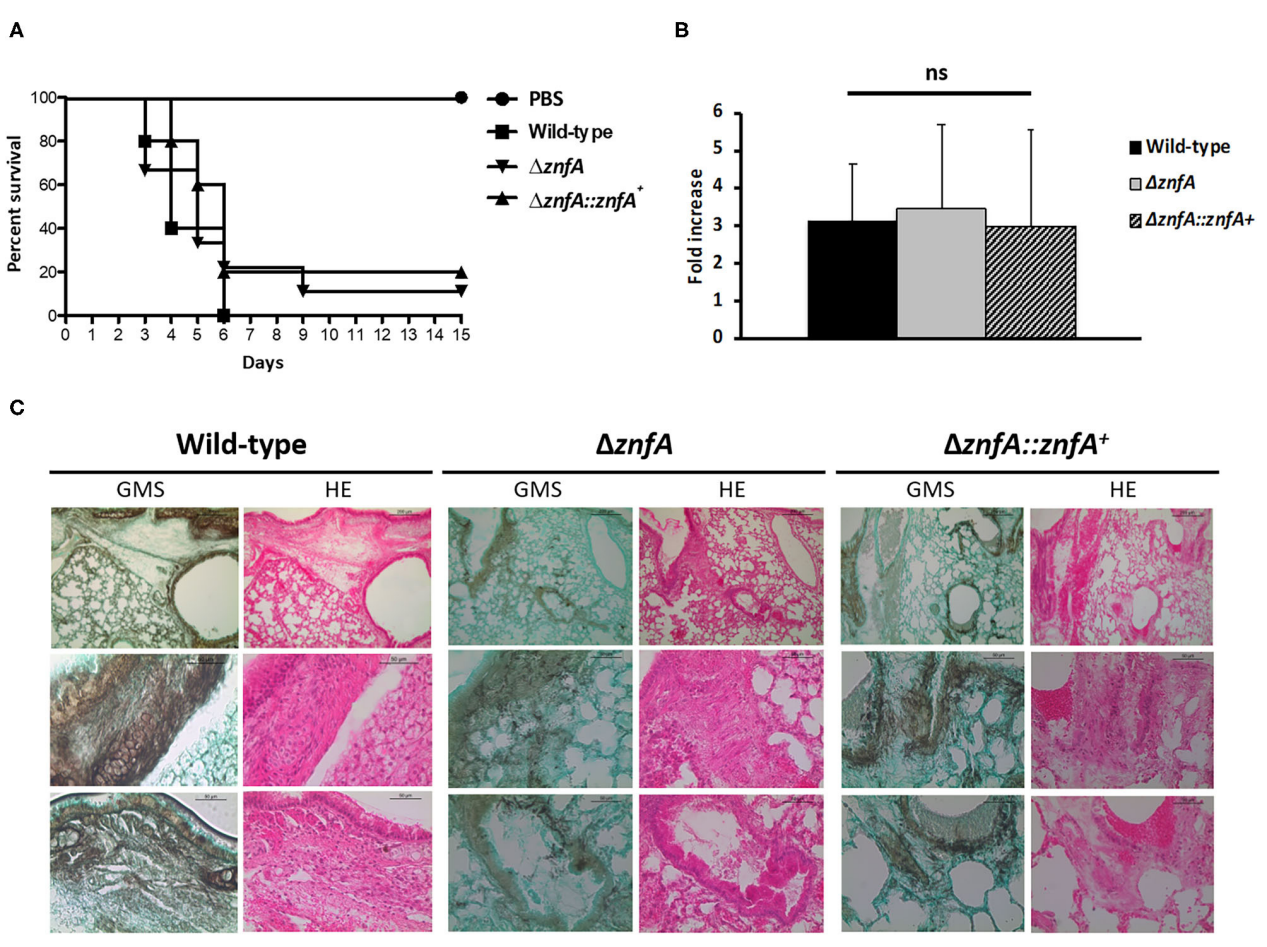
*A. fumigatus* ZnfA is not required for virulence in a chemotherapeutic murine model of invasive pulmonary aspergillosis. **(A)** Comparative survival analysis of Wild-type, Δ*znfA*, and Δ*znfA*::*znfA*^+^ strains in a chemotherapeutic murine model of invasive pulmonary aspergillosis. Mice in groups of 10 per strain were inoculated intranasally with a 20 μl suspension of conidia at a dose of 10^5^. PBS, Phosphate Buffer Saline. The statistical significance of comparative survival values was calculated by Prism statistical analysis package by using Log-rank (Mantel-Cox) Test and Gehan-Brestow-Wilcoxon tests. **(B)** Fungal burden was determined 48 h post-infection by real-time qPCR based on the 18S rRNA gene of *A. fumigatus* and an intronic region of the mouse GAPDH gene. Fungal and mouse DNA quantities were obtained from the Ct values from an appropriate standard curve. Fungal burden was determined through the ratio between ng of fungal DNA and mg of mouse DNA. The results are the means (±SD) of five lungs for each treatment. Statistical analysis was performed by using one-tailed, paired *t*-test (ns, not significant). **(C)** Histopathology of mice infected with *A. fumigatus* wild-type, Δ*znfA* or Δ*znfA*::*znfA*+ mutant strains. GMS (Grocott's Methenamine Silver) and HE (Hematoxylin and Eosin) staining of lung sections of representative of infections. The hatched area from the top row is magnified in the lower rows. Bars, 200 and 50 μm.

In order to evaluate the role of ZnfA in *A. fumigatus* virulence we used a chemotherapeutic mouse model of invasive pulmonary aspergillosis (Figure 6). Infection with the wild-type strain resulted in 100% mortality at 6 days post-infection, while infection with Δ*znfA* and Δ*znfA*::*znfA*^+^ strains resulted in 90 and 80% mortality at 15 days post-infection, respectively (Figure 6A). There was no statistical difference between survival curves of mice infected with the wild-type, Δ*znfA* and Δ*znfA*::znfA^+^ strains (*P* > 0.5 and *P* > 0.7; log rank, Mantel-Cox, and Gehan-Breslow-Wilcoxon tests, respectively). In addition, the fungal burden of lungs of mice infected with the three strains (Wild-type, Δ*znfA* and Δ*znfA*::*znfA*^+^) was measured by using qPCR, showing that all strains invaded the lung tissue similarly (*P* > 0.9; one-way ANOVA test) (Figure 6B). In agreement, multiple foci of invasive hyphal growth penetrating the pulmonary epithelium was observed in the histopathology of lungs of mice infected with the three strains (Figure 6C). Taken together, these results suggest that ZnfA is not important for *A. fumigatus* virulence.

## Discussion

Transcription factors (TFs) have long been recognized as key proteins in *A. fumigatus* regarding their involvement in fungal pathogenicity and virulence, but also in sensing environmental stress and antifungal resistance (Bultman et al., 2017; de Castro et al., 2019; Furukawa et al., 2020; Sturm et al., 2020; Valero et al., 2020). In this work we have characterized in detail ZnfA, a TF without any known functional annotation, that was identified in two previous phenotypic screenings of an *A. fumigatus* TF-null mutant library with high CaCl_2_ and CSP concentrations (de Castro et al., 2019; Valero et al., 2020). The link between calcium signaling and the cellular response to CSP has been already reported in *A. fumigatus*. High concentrations of CSP induce a spike in cytosolic calcium, activating calcineurin and consequently CrzA that directly binds to the promoter regions of genes encoding for chitin synthases (Fortwendel et al., 2010; Juvvadi et al., 2015; Ries et al., 2017). Moreover, CSP treatment increased the co-immunoprecipitation of proteins involved in cell wall biosynthesis and septation with calcineurin (Juvvadi et al., 2018). However, calcineurin-independent calcium responsive mechanisms are also involved in CPE regulation as demonstrated by ZipD transcriptional regulation of chitin synthase-encoding genes in the presence of the drug (Ries et al., 2017; de Castro et al., 2019).

The classical calcium-responsive factor CrzA translocates to the nucleus after being dephosphorylated by calcineurin as a result of different environmental stimuli (Soriani et al., 2008, 2010). However, although ZnfA exhibited nuclear localization after calcium and CSP exposure, it remained phosphorylated after calcium treatment, suggesting that phosphorylation does not play a direct role in the ZnfA post-translational regulation in the presence of calcium. Other post-translational modifications may be involved in the regulation of ZnfA. Alternatively, phosphorylation may be important for ZnfA under different conditions. This is in agreement with a recent report from our group, which showed that CN-dependent de-phosphorylation was not essential for ZipD nuclear translocation, another calcium and CSP-responsive TF (de Castro et al., 2019). We observed that the Δ*znfA* Δ*crzA* double deletion mutant is hypersensitive to calcium, cell wall and osmotic stresses when compared to the wild-type and single deletion mutants Δ*znfA* and Δ*crzA*. This synergistic phenotype can be explained due to some overlap between both TFs as we observed a 2-fold increase of ZnfA:3xHA binding to the CrzA promoter region in the presence of CSP. In contrast, the Δ*znfA* Δ*zipD* strain phenocopied the single deletion strain Δ*zipD*, except that it lost the CPE entirely and was slightly more sensitive to high CaCl_2_ concentrations, suggesting that ZnfA and ZipD may collaborate only in the response to calcium stress and during the CPE. We have determined that, upon CSP treatment, ZnfA also binds and modulates the expression of another TF, ZfpA. This gene has been reported to be induced in the presence of calcium and voriconazole and was identified in our previous screening with high CSP concentration (da Silva Ferreira et al., 2006; Soriani et al., 2010; Valero et al., 2020). Recently, ZfpA has been linked to oxylipin-induced hyphal branching in *A. fumigatus* (Niu et al., 2020).

We also know that ZnfA is involved in iron homeostasis in the presence of CSP by directly binding into the promoter regions of genes involved in the biosynthesis and transport of siderophores by blocking its expression and the loss of the CPE in the Δ*znfA* strain when grown in iron-depleted conditions. Siderophores are responsible for the uptake, transport and storage of iron in *A. fumigatus* cells and are essential for the full virulence of the fungus inside the host (Schrettl et al., 2004, 2007). The two classic transcriptional regulators of iron cellular levels in *A. fumigatus* are HapX and SreA. These TFs are interconnected in a negative feed-back loop as they repress each other maintaining iron homeostasis under control (Haas, 2014). However, their role has not been investigated yet in the presence of CSP. We have previously established a link between iron homeostasis and CPE in a mutant lacking the *fhdA* gene (Valero et al., 2020). Transcriptional analyses of this mutant in the presence of high concentrations of CSP revealed the upregulation of genes important for iron homeostasis, while the CPE was absent when this mutant was grown in iron-depleted media (Valero et al., 2020). Furthermore, several authors have reported synergistic antifungal effects of CSP and iron chelators (Lai et al., 2016; Sun et al., 2018; Bastos et al., 2019), thus reinforcing the idea that regulation of iron homeostasis is connected to the CSP cellular response. The ChIP-seq and gene expression analyses also showed that ZnfA directly regulates the expression of genes involved in cell wall organization. Chitin synthase genes *chsE, chsD* and *chs3* are directly controlled by ZnfA but only *chs3* has been directly linked to the CPE response in *A. fumigatus* (de Castro et al., 2019). Regarding β-1,3-glucan, ZnfA also promotes the expression of *fks1* in the presence of CSP, which encodes the catalytic subunit of the 1,3-β-glucan synthase (Beauvais et al., 2001), the CSP target, whose reactivation has been reported to be essential for the CPE (Loiko and Wagener, 2017). Furthermore, Sun1 and Sun2 have also been found to be involved in β-1,3-glucan modification and important for cell wall biogenesis (Gastebois et al., 2013). In agreement with this, we found that cell wall extracts of the Δ*znfA* and Δ*znfA* Δ*zipD* strains exhibited lower glucose concentrations than the wild-type and complemented strains, while the opposite was observed for the Δ*znfA* Δ*crzA* strain, confirming the role of ZnfA in the biosynthesis and modification of cell wall β-1,3-glucan. GflA and GlfB promoter regions were also bound by ZnfA after CSP exposure, these proteins are involved in the synthesis and transport of galactofuranose, a cell wall component important for virulence and antifungal resistance in *A. fumigatus* (Schmalhorst et al., 2008; Engel et al., 2009). Despite the observation that the architecture and cell wall polysaccharide concentrations in the strains included in this work differed from the wild-type strain, none of our mutant strains exhibited increased susceptibility to any antifungal drug tested. It also remains to be determined if cell wall alterations caused by the deletion of *znfA* influence recognition by immune cells and subsequent activation of the immune system. However, survival curve analysis, fungal burden determination and histopathology results indicate that *znfA* is not required for *A. fumigatus* virulence in a chemotherapeutic mouse model of invasive pulmonary aspergillosis.

In summary, we have characterized in detail ZnfA, a newly identified transcription factor involved in calcium-responsive and CSP tolerance mechanisms. This TF mainly acts by directly regulating iron homeostasis and cell wall composition and organization in the presence of CSP while collaborates with CrzA in the response to calcium, cell wall and osmotic stresses. This work advances our understanding of the link between calcium signaling and CSP cellular responses thus revealing complex connections between different stress sensing pathways in *A. fumigatus*.

## Materials and Methods

### Strains and Media

Strains were grown at 37°C in either complete medium (YAG) [2% (w/v) glucose, 0.5% (w/v) yeast extract, trace elements] or minimal medium (MM) [1% (w/v) glucose, nitrate salts, trace elements, pH 6.5]. Solid YAG and MM were the same as described above except that 1.7% (w/v) or 2% (w/v) agar was added. Trace elements, vitamins, and nitrate salts compositions were as described previously (Käfer, 1977). For iron limitation experiments, strains were growth in solid MM without iron (MM-Fe) [containing glucose 1% (wt/vol), trace elements without FeSO_4_, agar 2% (wt/vol)]. Wherever required, MM was supplemented at stated concentrations with calcium chloride (CaCl_2_), caspofungin (CSP), Congo red (CR), calcofluor white (CFW), sorbitol, sodium chloride (NaCl), ethylene glycol tetraacetic acid (EGTA), or FeSO_4_. For phenotype characterization, plates were inoculated with 10^4^ spores per strain and left to grow for 120 h at 37 or 30°C. All radial growths were expressed as ratios, dividing colony radial diameter of growth in the stress condition by colony radial diameter in the control (no stress) condition.

### Construction of *A. fumigatus* Strains

Δ*znfA* and Δ*zipD* strains belong to a TF deletion mutant library reported in Furukawa et al. (2020) and were constructed as described in Zhao et al. (2019). Δ*znfA::znfA*^+^ complemented strain was obtained by co-transformation of the *znfA* ORF amplified from *A. fumigatus* CEA17 genomic DNA (gDNA) with znfA_ext_F and znfA_ext_R primers and the plasmid pPRTI containing the gene that confers resistance to pyrithiamine. MM supplemented with pyrithiamine (1 μg/ml) was used as selective media for positive transformants.

Δ*crzA*, Δ*crzA::crzA*^+^ and Δ*zipD*::*zipD*^+^ strains were already described in Ries et al. (2017) and de Castro et al. (2019).

ZnfA:GFP and ZnfA:3xHA protein fusion constructions and Δ*znfA* Δ*crzA* and Δ*znfA* Δ*zipD* double deletion mutants were obtained by *in vivo* recombination in *Saccharomyces cerevisiae*, as described in Malavazi and Goldman (2012). Approximately, 1 kb from the 5′-UTR and 3′-UTR flanking region of the target gene was selected for primer design. The primers pRS426_UTR5′_F and pRS426_UTR3′_R contain a short sequence homologous to the multiple cloning site of the pRS426 plasmid. Both the 5′ and 3′ UTR fragments were PCR-amplified from *A. fumigatus* CEA17 genomic DNA. For ZnfA:3xHA construction, the 3xHA-trpC-pyrG fragment was amplified from the pOB430 plasmid, while for ZnfA:GFP construction, the GFP-trpC-prtA fragment was amplified from a previously constructed strain. For Δ*znfA* Δ*crzA* and Δ*znfA* Δ*zipD*, the *prtA* gene was used as a prototrophy marker in the deletion cassettes and was amplified from the pPRTI plasmid. Gene replacement cassettes were amplified directly from yeast gDNA using TaKaRa Ex Taq DNA Polymerae (Clontech Takara Bio) and were transformed into protoplasts of the *A. fumigatus* ΔakuBKU80 pyrG- or Δ*znfA* strains according to standard protocols (Malavazi and Goldman, 2012). MM or MM supplemented with pyrithiamine (1 μg/ml) was used for selective media for positive transformants.

Growth phenotypes in the presence of selection drugs were performed in order to check strain functionality (Supplementary Figure 2). Southern blot and PCR analyses were used to verify homologous cassette integration in *A. fumigatus* genome (Supplementary Figure 3). All primers used for strain constructions and verification are listed in Supplementary Table 3.

### Microscopy

ZnfA:GFP protein fusion strain conidiospores were grown on coverslips in 5 ml of liquid MM for 16 h at 30°C. After incubation, coverslips with adherent germlings were left untreated or treated CaCl_2_ (200 mM) for 10 and 30 min or caspofungin (0,125 μg/ml) for 10 min, following with Hoechst 33342 dye (Molecular Probes, Eugene, OR, USA) (20 μg/ml) for 10 min. Subsequently, the coverslips were rinsed with phosphate-buffered saline (PBS; 140 mM NaCl, 2 mM KCl, 10 mM NaHPO_4_, 1.8 mM KH_2_PO_4_, pH 7.4) and mounted for examination. Slides were visualized on an Observer Z1 fluorescence microscope using a 100x objective oil immersion lens. For GFP condition, filter set 38-high efficiency (HE) was used with excitation wavelength of 450–490 nm and emission wavelength of 500–550 nm, while for Hoechst/DAPI [4′,6-diamidino-2-phenylindole] stain, filter set 49 was used with excitation wavelength of 365 nm and emission wavelength of 420–470 nm. DIC (differential interference contrast) images and fluorescent images were captured with an AxioCam camera (Carl Zeiss, Oberkochen, Germany) and processed using AxioVision software (version 4.8).

### Bidimensional Electrophoresis and Immunoblot Analysis

To assess protein phosphorylation status of ZnfA, conidia (1 × 10^8^) of the ZnfA:3xHA strain were inoculated in 50 ml liquid MM at 37°C for 24 h and left untreated or treated with CaCl_2_ 10 mM for 10 min. Mycelia were frozen with liquid nitrogen, ground, and 500 mg were resuspended in 1 ml of B250 buffer. Samples were centrifuged at maximum speed for 10 min at 4°C. The supernatant was removed, and a Bradford assay (BioRad) was carried out to measure protein content. The same amount of protein for each sample was added to 20 μl of Dynabeads Protein A (Thermo Fisher Scientific) previously incubated with monoclonal anti-HA antibody (Sigma). The beads were washed three times with resuspension buffer prior to incubation. Cell extracts and resin were incubated with shaking at 4°C for 2 h. After incubation, the beads were washed three times with the resuspension buffer by placing the tube in a DynaMag™ magnet. For the lambda protein phosphatase treatment, the ZnfA:3xHA immunoprecipitates bound to magnetic beads were incubated with PMP buffer (50 mM HEPES pH 7.5, 100 mM NaCl, 2 mM DTT, 0.01% Brij 35, and 1 mM MnCl_2_) and 400 units of lambda protein phosphatase (New England BioLabs) (without phosphatase inhibitor cocktail) for 1 h at 30°C. Proteins were finally released by the beads by the addition of Rehydration buffer (7 M Urea, 2 M Thiourea, 2% CHAPS, 0.35 mg/sample DTT). The isoelectric focalization (IEF) was performed using Ettan IPGphor 3 System (GE Healthcare), with 7 cm DryStrip (pH 3–10) immobiline strips. The IEF was carried out following the default steps suggested in GE Healthcare 2D instructions (300 V for over 200 Vh; ramping 1,000 V for over 300 Vh and 5,000 V for 4,000 Vh; holding at 5,000 V for 3,000 Vh). The second dimension was performed by incubation of the strips with 1% DTT in equilibration buffer (75 mM Tris-HCl pH 8.8; 6 M urea; 29% glycerol and 2% SDS) for 15 min followed by 15 min in equilibration buffer with 2.5% iodoacetamide. The strips were washed in water and transferred to the top of 12% SDS-Polyacrylamide gel, covered with 0.5% agarose solution and submitted to electrophoresis. After running, the proteins from the gels were transferred to a nitrocellulose membrane for a western blot assay. To detect ZnfA proteins, a monoclonal Anti-HA antibody produced in mouse (Sigma) was used. Primary antibody was detected using an HRP-conjugated secondary antibody raised in mouse (Sigma). Chemoluminescent detection was achieved using an ECL Prime Western Blot detection kit (GE HealthCare) following manufacturer's instructions.

### Chromatin Immunoprecipitation Coupled to DNA Sequencing (ChIP-seq)

1 × 10^8^ conidia of the ZnfA:3xHA strain were grown into 100 ml of liquid minimal media for 16 h at 37°C under shaking conditions before 2 μg/ml of CSP was added for 1 h. The DNA was crosslinked by adding 3 ml of formaldehyde 37% to the culture media with gentle rocking for 20 min at room temperature. To stop the reaction, glycine was added to a final concentration of 1 M and incubated for additional 10 min. Mycelia were filtered using miracloth, washed twice with cold water, frozen with liquid nitrogen and dried. Fifteen milligram of dry mycelia were lysed in 800 μl of FA lysis buffer for 3 min using a Bullet Blender (Next Advance), resuspended in 500 μl of FA lysis buffer and sonicated using the Qsonica Q800R at 100% amplitude with 10 s ON and 15 s OFF cycles for a total sonication time of 30 min. Approximately 2 μg of the chromatin recovered immuno-precipitated using anti-HA (HA) antibody (F7, Santa Cruz Biotechnology) for 1.5 h, followed by incubation with ~15 μl of packed Protein A Sepharose matrix (GE Healthcare) for another 1.5 h at room temperature. The Protein A beads were washed with increasing ionic strength solutions to remove the unspecific bindings. The DNA was eluted and de-crosslinked at 65°C overnight. The purified DNA was used for library preparation using the NEBNext® Ultra II Library Prep kit (Illumina, cat. no. E7645L) according to manufacturer's protocol. The libraries were mixed in an equal molar ratio and sequenced using the Illumina HiSeq2500 platform at the Genomics and Single Cells Analysis Core facility at the University of Macau.

### ChIP-Seq Analysis

For ZnfA:3xHA binding sites analysis, the raw sequencing reads mapping and peak calling were performed as described previously (Ries et al., 2020). The peaks located up to 2,000 base pairs (bp) from the coding region were selected for further analysis following the subsequent parameters: pileUp > 29; *p*-value > 3.5; fold enrichment > 1.7; *q*-value > 1.7. To ensure significance, the peaks were manually checked using Integrative genomics viewer (IGV) tool (Robinson et al., 2011). For motif enrichment analysis 500 bp sequences around the peak summit of the strongest peaks [pileUp > 50; fold_enrichment > 2; log10(*P*-value) > 6.5; log10(*Q*-value) > 4] extracted from Af293 genome were used. For MEME-ChIP analysis (Machanick and Bailey, 2011), the classic discovery mode was applied to search for the motifs in JASPER database. The ChIP-seq data are available from NCBI SRA (sequence read archive) database under accession numbers GSM5113978-81.

### RNA Extraction and RT-qPCR Analysis

1 × 10^7^ conidia of wild-type and Δ*znfA* strains were grown in MM for 20 h at 37°C under shaking conditions following the addition of 2 μg/ml of CSP. Untreated mycelia were used as controls, thus mimicking the ChIP-seq experimental design. RNA was extracted by using TRIzol reagent (Invitrogen) following DNA digestion with RQ1 RNase-free DNase (Promega) according to the manufacturer's instructions. Total RNA was reverse-transcribed into cDNA by using an ImProm-II reverse transcription system (Promega) and oligo (dT). The amplification assay was carried out in a 7500 real-time PCR system (Applied Biosystems). qPCRs were performed in a 10-μl final volume containing Sybr green PCR master mix (Applied Biosystems) under the following conditions: an initial step of 2 s at 50°C, followed by 10 min at 95°C and 40 cycles at 95°C for 15 s and 60°C for 1 min. Three independent biological replicates were used, and mRNA quantity relative fold change data were calculated using standard curves and normalized by α-actin expression as described in Valero et al. (2020). All the primers used for RT-qPCR experiments are described in Supplementary Table 3.

### Cell Wall Polysaccharides Extraction and Sugar Quantification

Fungal cell wall polysaccharides were extracted from 100 mg dry-frozen biomass as described previously using TFA hydrolysis (Trichloroacetic acid) hydrolysis (Dallies et al., 1998). Total carbohydrates were estimated using phenol sulfuric method as described by Masuko et al. (2005). Released sugars from hydrolysis were subsequently analyzed by high-performance liquid chromatography (HPLC) using a YoungLin YL9100 series system (YoungLin, Anyang, Korea) equipped with a YL9170 series refractive index (RI) detector at 40°C. Samples were loaded in a REZEX ROA (Phenomenex, USA) column (300 × 7.8 mm) at 85°C and eluted with 0.05 M sulfuric acid at a flow rate of 0.5 ml/min. All sugars concentrations were expressed in millimolar (mM) using a correspondent standard curve.

### Transmission Electron Microscopy Analysis of Cell Wall

1 × 10^7^ conidia of the relevant strains were grown statically at 37°C in MM for 24 h. Mycelia were harvested and immediately fixed in 0.1 M sodium phosphate buffer (pH 7.4) containing 2.5% (v/v) of glutaraldehyde and 2% (w/v) of paraformaldehyde for 24 h at 4°C. Samples were encapsulated in agar (2% w/v) and subjected to fixation (1% OsO_4_), contrasting (1% uranyl acetate), ethanol dehydration, and a two-step infiltration process with Spurr resin (Electron Microscopy Sciences) of 16 and 3 h at room temperature. Additional infiltration was provided under vacuum at room temperature before embedment in BEEM capsules (Electron Microscopy Sciences) and polymerization at 60°C for 72 h. Semithin (0.5-μm) survey sections were stained with toluidine blue to identify the areas of best cell density. Ultrathin sections (60 nm) were prepared and contrasted again with uranyl acetate (1%) and lead citrate (2%). TEM images were obtained using a Philips CM-200 electron microscope at an acceleration voltage of 120 kV using a MegaView3 camera. Cell wall thickness of 100 sections of different germlings were measured and images analyzed with the ImageJ software. Statistical differences were evaluated by using one-way ANOVA with Dunnett's multiple comparison test.

### Determination of Minimal Inhibitory Concentrations

Strains were grown in 96-well plates at a concentration of 10^4^ spores/well in 200 μl of MM supplemented with increasing concentrations of amphotericin B, voriconazole, itraconazole, and posaconazole, according to the protocol elaborated by the Clinical and Laboratory Standards Institute (CLSI, 2017). Three independent experiments including two technical replicates for each strain were carried out for each antifungal drug.

### Chemotherapeutic Murine Model of Invasive Pulmonary Aspergillosis

Outbreed female mice (BALB/c strain; body weight, 20–22 g) were housed in vented cages containing 5 animals. Mice were immunosuppressed with cyclophosphamide (150 mg/kg of body weight) administered intraperitoneally on days −4, −1, and 2 prior to and post infection. Hydrocortisonacetate (200 mg/kg of body weight) was injected subcutaneously on day −3. *A. fumigatus* conidia of Δ*znfA*, Δ*znfA::znfA*^+^ and wild-type strains were grown in YAG plates for 2 days prior to infection and were harvested in PBS and filtered through a Miracloth (Calbiochem). Conidial suspensions were washed three times with PBS, counted using a hemocytometer, and resuspended at a concentration of 5 × 10^6^ conidia/ml. The viability of the administered inoculum was determined by incubating a serial dilution of the conidia on YAG medium, at 37°C. Mice were anesthetized by halothane inhalation and infected by intranasal instillation of 10^5^ conidia in 20 μl of PBS. As a negative control, a group of 5 mice received PBS only. Mice were weighed every 24 h from the day of infection and visually inspected twice daily. The statistical significance of the comparative survival values was calculated using log rank analysis and the Prism statistical analysis package.

To assess fungal burden and histopathology in the lungs, mice were infected as described previously, but with a higher inoculum (10^6^ conidia/20 μl) to increase fungal DNA detection. Animals were sacrificed 72 h post-infection, and both lungs were harvested and immediately frozen in liquid nitrogen for fungal burden determination or fixed for 24 h in 3.7% formaldehyde-PBS for histopathology analysis.

Samples for fungal burden determination were processed as described in (de Castro et al., 2019). The lungs were homogenized by vortexing with glass beads for 10 min and DNA was extracted *via* the phenol-chloroform method. DNA quantity and quality were assessed using a NanoDrop 2000 spectrophotometer (Thermo Scientific). At least 500 ng of total DNA from each sample was used for qPCR that were carried out as described before. Six-point standard curves were calculated using serial dilutions of gDNA from all the *A. fumigatus* strains used and the uninfected mouse lung. Fungal and mouse DNA quantities were obtained from the threshold cycle values from an appropriate standard curve. Fungal burden was determined as the ratio between fungal and mouse DNA and expressed as fold increase (de Castro et al., 2019).

Additionally, samples for histopathology analysis were washed several times in 70% alcohol before dehydration in a series of alcohol solutions at increasing concentrations. Finally, the samples were incubated in xylol and embedded in paraffin. For each sample, sequential 5 μm-thick sections were collected on glass slides and stained with Gomori methenamine silver (GMS) or hematoxylin and eosin (HE) stains following standard protocols (Greenberger, 2002). Briefly, the sections were deparaffinized, oxidized with 4% chromic acid, stained with methenamine silver solution, and counterstained with hematoxylin. Tissue sections were also stained with hematoxylin and eosin for histological examination to determine lung damage. All the stained slides were immediately washed, preserved in mounting medium, and sealed with a coverslip. Microscopic analyses were performed using an Axioplan 2 imaging microscope (Carl Zeiss) at the stated magnifications under bright-field conditions.

The principles that guide our studies are based on the Declaration of Animal Rights ratified by the UNESCO in January 27, 1978 in its 8th and 14th articles. All protocols adopted in this study were approved by the local ethics committee for animal experiments from the University of São Paulo, Campus of Ribeirão Preto (Permit Number: 08.1.1277.53.6; Studies on the interaction of *Aspergillus fumigatus* with animals). Groups of five animals were housed in individually ventilated cages and were cared for in strict accordance with the principles outlined by the Brazilian College of Animal Experimentation (COBEA) and Guiding Principles for Research Involving Animals and Human Beings, American Physiological Society. All efforts were made to minimize suffering. Animals were clinically monitored at least twice daily and humanely sacrificed if moribund (defined by lethargy, dyspnea, hypothermia, and weight loss). All stressed animals were sacrificed by cervical dislocation.

## Data Availability Statement

The datasets presented in this study can be found in online repositories. The names of the repository/repositories and accession number(s) can be found at: NCBI GEO (accession: GSE167847).

## Ethics Statement

The animal study was reviewed and approved by the local Ethics Committee for animal experiments from the University of São Paulo, Campus of Ribeirão Preto (Permit Number: 08.1.1277.53.6; Studies on the interaction of *Aspergillus fumigatus* with animals).

## Author Contributions

GG and CV conceived and designed the experiments. CV, AC, PdC, LS, LR, MR, IM, RS, CM, and PD performed the experiments. CV, GG, AC, LP, FW, MR, and IM analyzed the data. GG, IM, RS, CP-S, MB, and KW contributed with reagents, materials, and analysis tools. CV drafted the manuscript. GG, AC, LR, MR, IM, LP, CP-S, and KW critically revised the manuscript. All authors contributed to the article and approved the submitted version.

## Conflict of Interest

The authors declare that the research was conducted in the absence of any commercial or financial relationships that could be construed as a potential conflict of interest.

## Publisher's Note

All claims expressed in this article are solely those of the authors and do not necessarily represent those of their affiliated organizations, or those of the publisher, the editors and the reviewers. Any product that may be evaluated in this article, or claim that may be made by its manufacturer, is not guaranteed or endorsed by the publisher.

## Supplementary Material

Supplementary Figure 1 Radial growth determination of *A. fumigatus* strains included in this work in the presence of increasing concentrations of **(A,C)** the calcium chelant agent EGTA and **(B,D)** at 30°C. Standard deviations represent averages of results from three independent biological repetitions. Statistical analysis was performed using one-tailed, paired *t*-tests for comparisons to the control condition (^*^*P* < 0.05; ^**^*P* < 0.01; ^****^*P* < 0.0001).

Supplementary Figure 2 **(A)** Radial growth determination of *A. fumigatus* ZnfA:3xHA and ZnfA:GFP strains under key stressors in order to check strains functionality (ns, not significant). **(B)** Growth phenotypes in the presence of selection drugs were performed in order to check functionality of strains constructed in this work. **(C)** Number of conidia in the wild-type, Δ*znfA*, Δ*crzA*, Δ*znfA*Δ*crzA* and complemented strains. Freshly harvested conidia (1 × 10^4^) of aforementioned strains were inoculated onto solid MM at 37°C for 5 days. Four circular sections of ~1 cm^2^ were taken from each plate and placed in a falcon tube containing 10 ml of a 0.01% tween solution. Conidia were counted after intensive vortexing using a Neubauer chamber. The assay was performed in triplicate. Standard deviations represent averages of results from three independent biological repetitions. Statistical analysis was performed using one-tailed, paired *t*-tests for comparisons to the control condition (^****^*P* < 0.0001).

Supplementary Figure 3 **(A)** Southern blot analysis of wild-type, Δ*znfA*, and Δ*znfA*::*znfA*^+^ strains used to verify homologous cassette integration in the complemented strain. **(B)** PCR analyses used to verify homologous cassette integration of *znfA* locus related constructions in Δ*znfA*Δ*crzA*, Δ*znfA*Δ*zipD*, ZnfA:3xHA and ZnfA:GFP strains. **(C)** PCR analyses used to verify homologous cassette integration of *crzA* locus related construction in Δ*znfA*Δ*crzA* strain. **(D)** PCR analyses used to verify homologous cassette integration of *zipD* locus related construction in Δ*znfA*Δ*zipD* strain.

Supplementary Table 1 Binding of ZnfA:3xHA to target genes that are shared between the control and caspofungin conditions (common), unique to the control condition (control_exclusive) and unique to caspofungin condition (casofungin_exclusive).

Supplementary Table 2 MIC values and ranges (μg/ml) obtained by CLSI antifungal susceptibility testing method for the *A. fumigatus* strains included in this work.

Supplementary Table 3 List of primers used in this work.
