# Supplementary figures and images for: Aspergillus Fumigatus ZnfA, a Novel Zinc Finger Transcription Factor Involved in Calcium Metabolism and Caspofungin Tolerance
Source: Front Fungal Biol. 2021 Aug 10;2:689900. doi: 10.3389/ffunb.2021.689900 (PMC10512341; doi:10.3389/ffunb.2021.689900)

A.

MM (30°C)

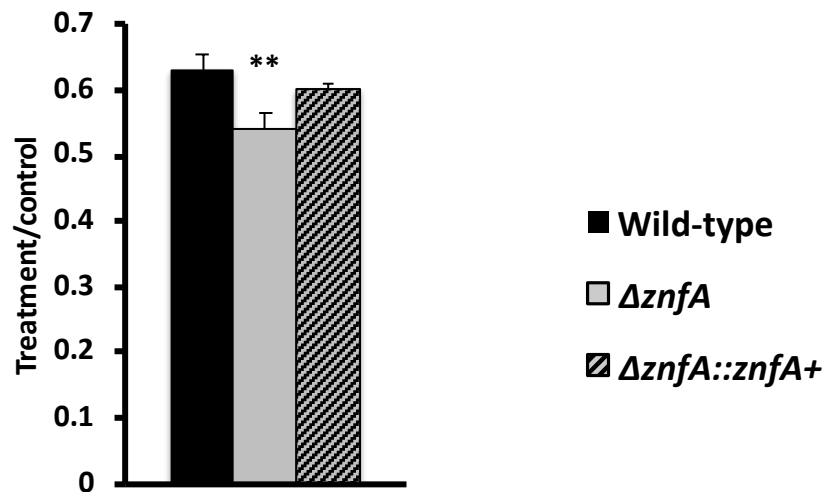

B.

EGTA (mM)

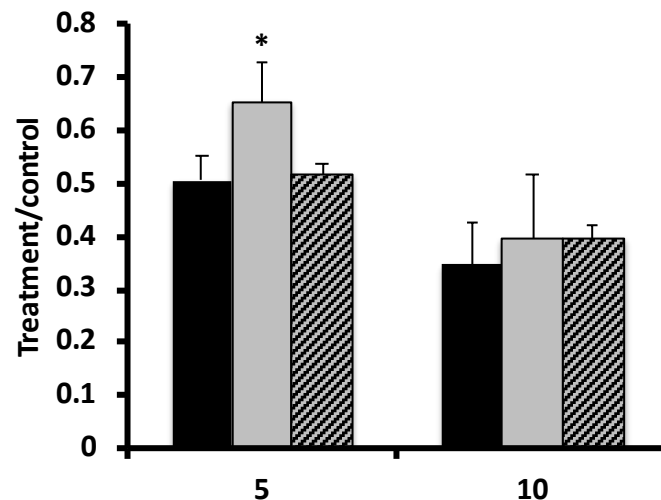

C.

MM (30°C)

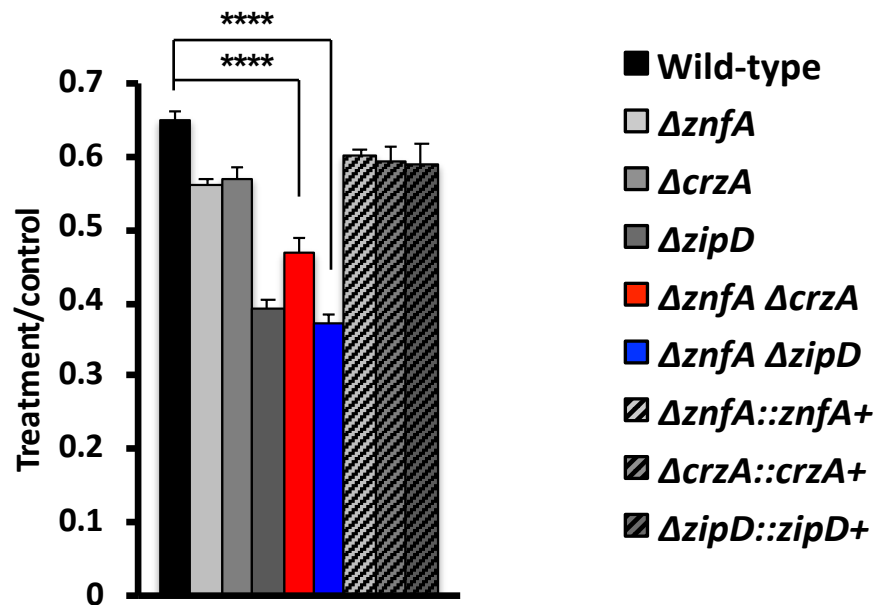

D.

EGTA (mM)

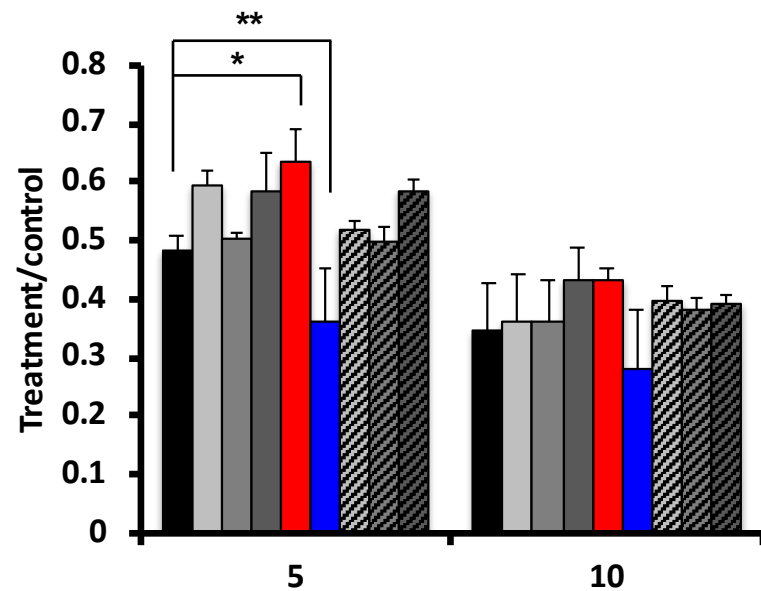

Supplement: Supplementary Figure 1 — Radial growth determination of A. fumigatus strains included in this work in the presence of increasing concentrations of (A,C) the calcium chelant agent EGTA and (B,D) at 30°C. Standard deviations represent averages of results from three independent biological repetitions. Statistical analysis was performed using one-tailed, paired t-tests for comparisons to the control condition (*P < 0.05; **P < 0.01; ****P < 0.0001). [file Data_Sheet_1.PDF]

A.

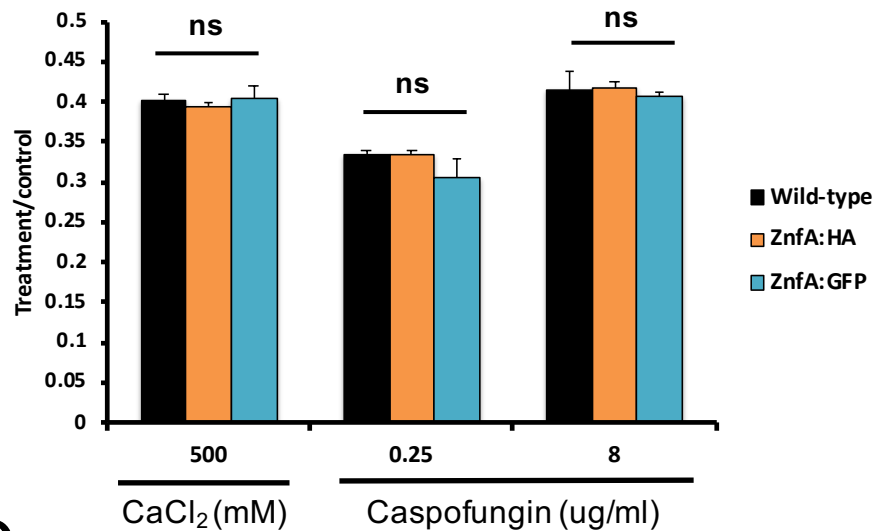

B.

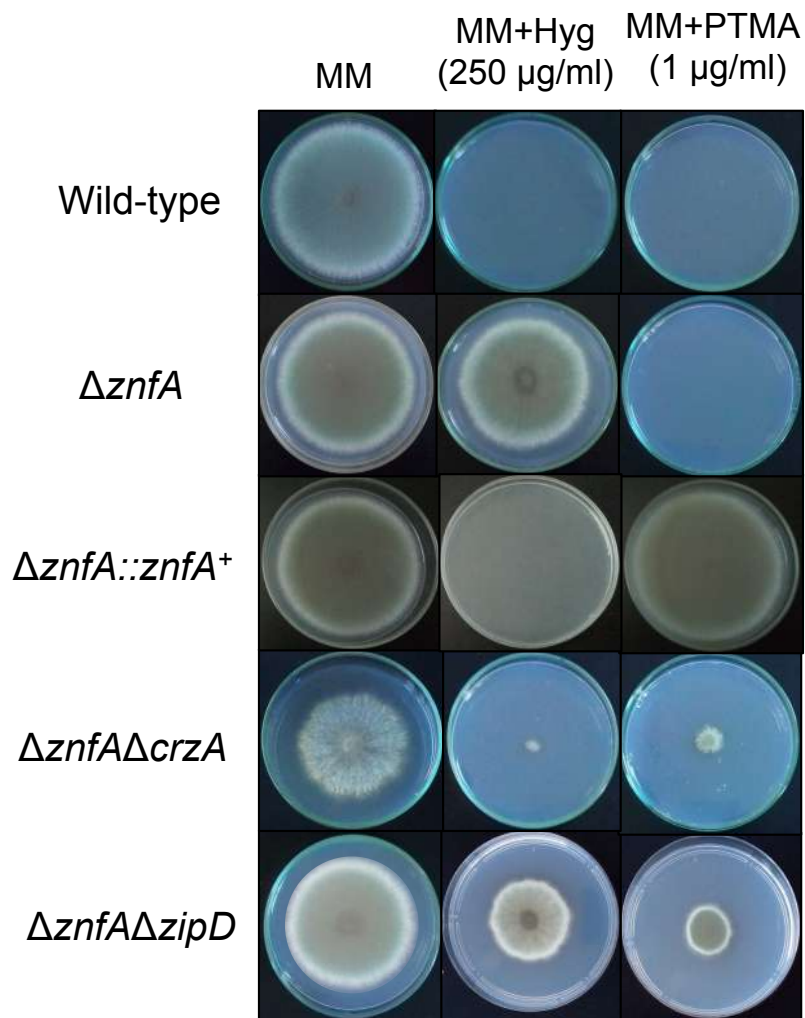

C.

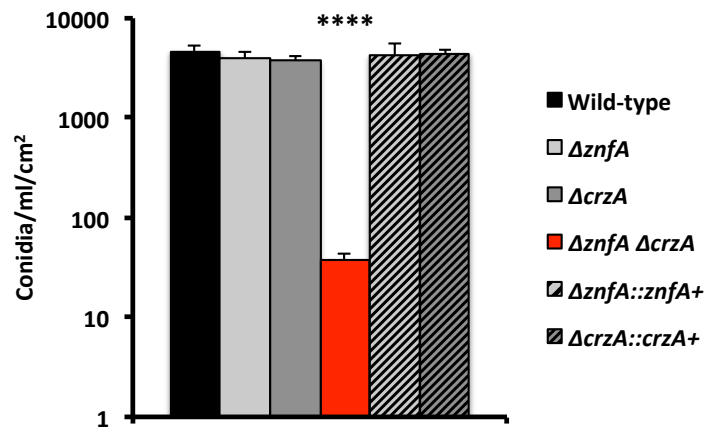

Supplement: Supplementary Figure 2 — (A) Radial growth determination of A. fumigatus ZnfA:3xHA and ZnfA:GFP strains under key stressors in order to check strains functionality (ns, not significant). (B) Growth phenotypes in the presence of selection drugs were performed in order to check functionality of strains constructed in this work. (C) Number of conidia in the wild-type, ΔznfA, ΔcrzA, ΔznfAΔcrzA and complemented strains. Freshly harvested conidia (1 × 10^4^) of aforementioned strains were inoculated onto solid MM at 37°C for 5 days. Four circular sections of ~1 cm2 were taken from each plate and placed in a falcon tube containing 10 ml of a 0.01% tween solution. Conidia were counted after intensive vortexing using a Neubauer chamber. The assay was performed in triplicate. Standard deviations represent averages of results from three independent biological repetitions. Statistical analysis was performed using one-tailed, paired t-tests for comparisons to the control condition (****P < 0.0001). [file Data_Sheet_2.PDF]
