# Supplementary material for: Aspergillus Fumigatus ZnfA, a Novel Zinc Finger Transcription Factor Involved in Calcium Metabolism and Caspofungin Tolerance
Source: Front Fungal Biol. 2021 Aug 10;2:689900. doi: 10.3389/ffunb.2021.689900 (PMC10512341; doi:10.3389/ffunb.2021.689900)
Supplement: Supplementary Figure 3 — (A) Southern blot analysis of wild-type, ΔznfA, and ΔznfA::znfA+ strains used to verify homologous cassette integration in the complemented strain. (B) PCR analyses used to verify homologous cassette integration of znfA locus related constructions in ΔznfAΔcrzA, ΔznfAΔzipD, ZnfA:3xHA and ZnfA:GFP strains. (C) PCR analyses used to verify homologous cassette integration of crzA locus related construction in ΔznfAΔcrzA strain. (D) PCR analyses used to verify homologous cassette integration of zipD locus related construction in ΔznfAΔzipD strain. [file Data_Sheet_3.PDF]

A.

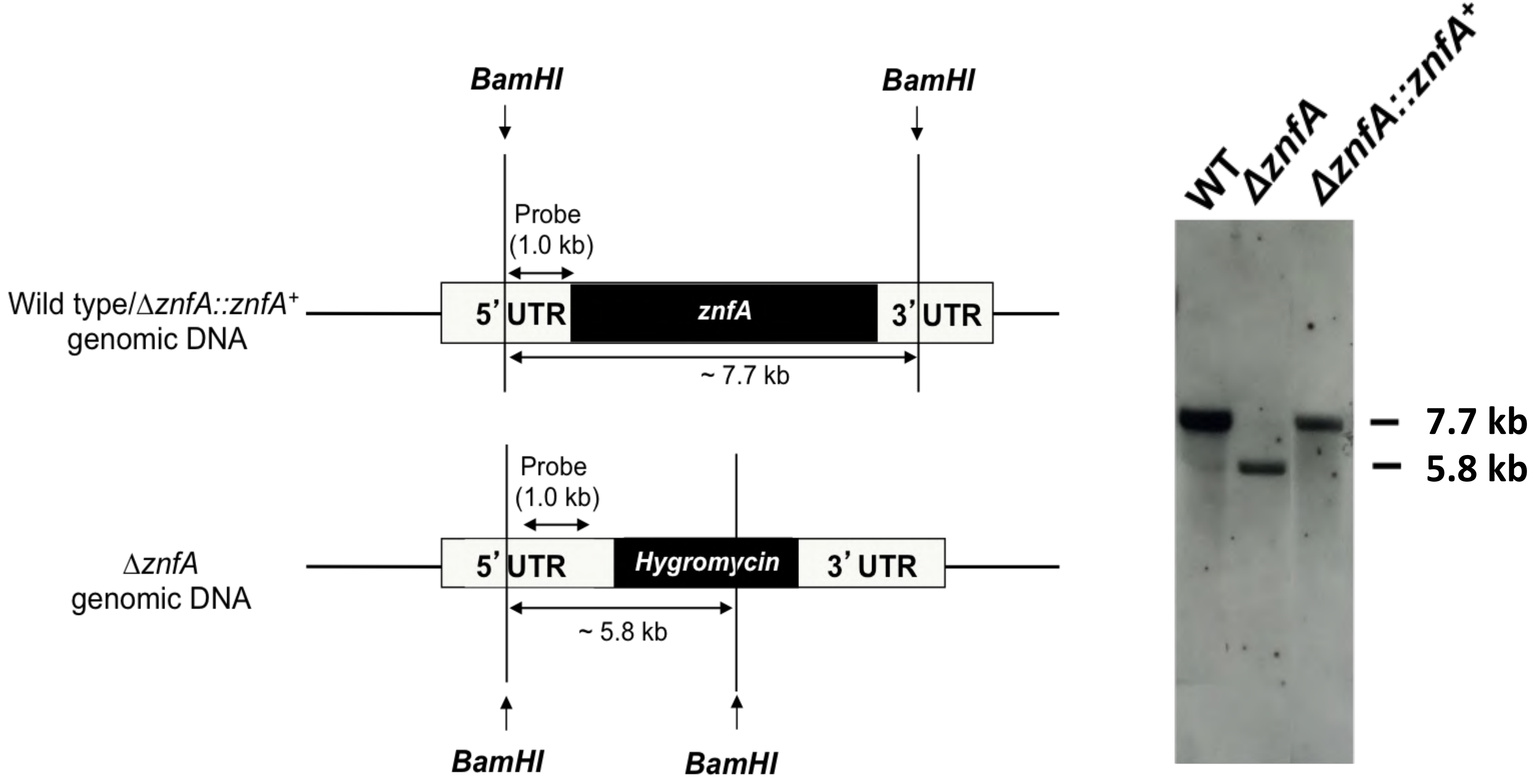

BamHI
BamHI
Probe
(1.0 kb)
Wild type/ΔznfA::znfA+
genomic DNA
5' UTR
znfA
3' UTR
~ 7.7 kb
Probe
(1.0 kb)
ΔznfA
genomic DNA
5' UTR
Hygromycin
3' UTR
~ 5.8 kb
BamHI
BamHI
WT
ΔznfA
ΔznfA::znfA+
7.7 kb
5.8 kb

B.

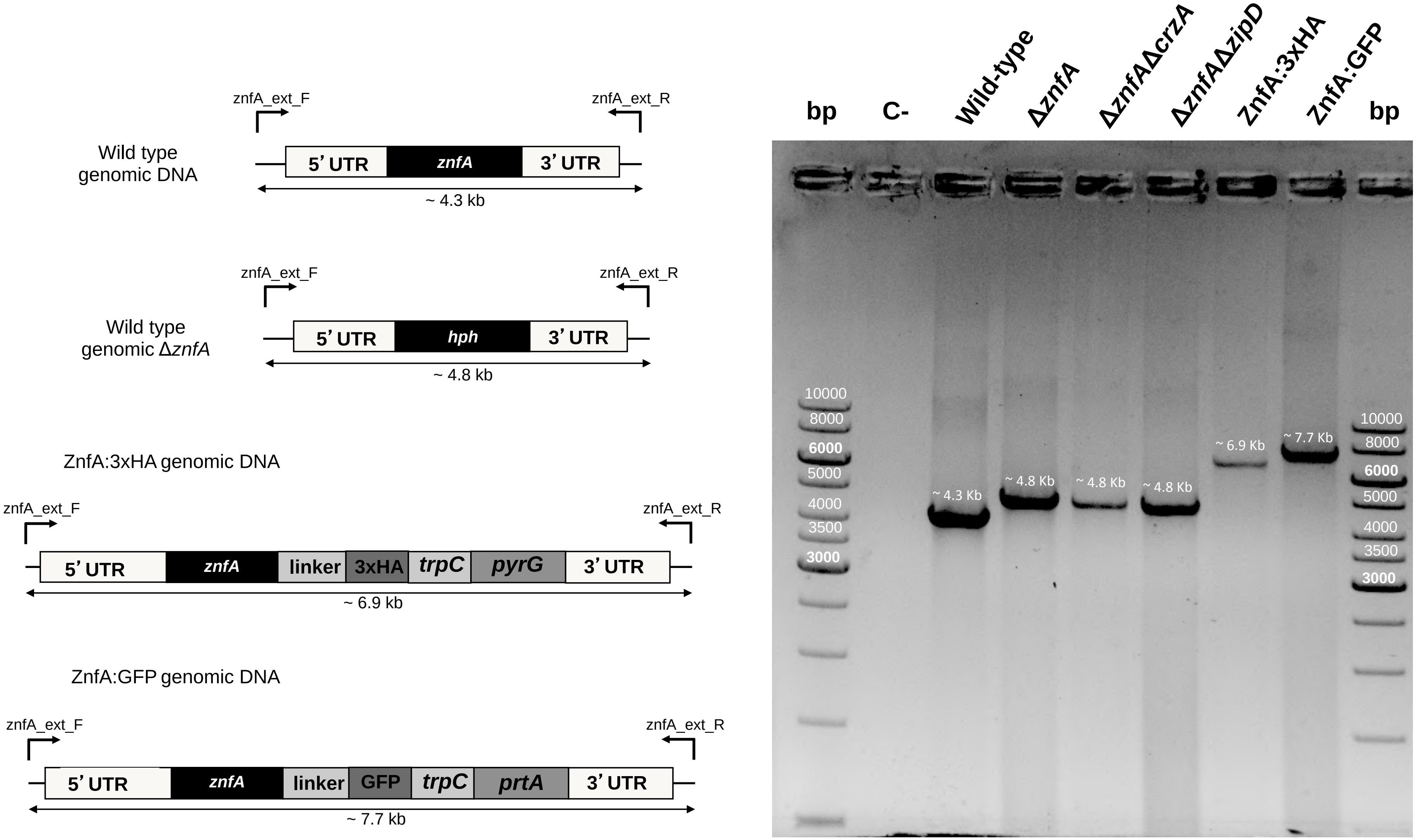
znfA_ext_F
znfA_ext_R
Wild type genomic DNA
5' UTR
znfA
3' UTR
~ 4.3 kb
znfA_ext_F
znfA_ext_R
Wild type genomic ΔznfA
5' UTR
hph
3' UTR
~ 4.8 kb
ZnfA:3xHA genomic DNA
znfA_ext_F
znfA_ext_R
5' UTR
znfA
linker
3xHA
trpC
pyrG
3' UTR
~ 6.9 kb
ZnfA:GFP genomic DNA
znfA_ext_F
znfA_ext_R
5' UTR
znfA
linker
GFP
trpC
prtA
3' UTR
~ 7.7 kb
bp
C-
Wild-type
ΔznfA
ΔznfAΔcrzA
ΔznfAΔzipD
ZnfA:3xHA
ZnfA:GFP
bp
10000
8000
6000
5000
4000
3500
3000
~ 4.3 Kb
~ 4.8 Kb
~ 4.8 Kb
~ 4.8 Kb
~ 6.9 Kb
~ 7.7 Kb
10000
8000
6000
5000
4000
3500
3000

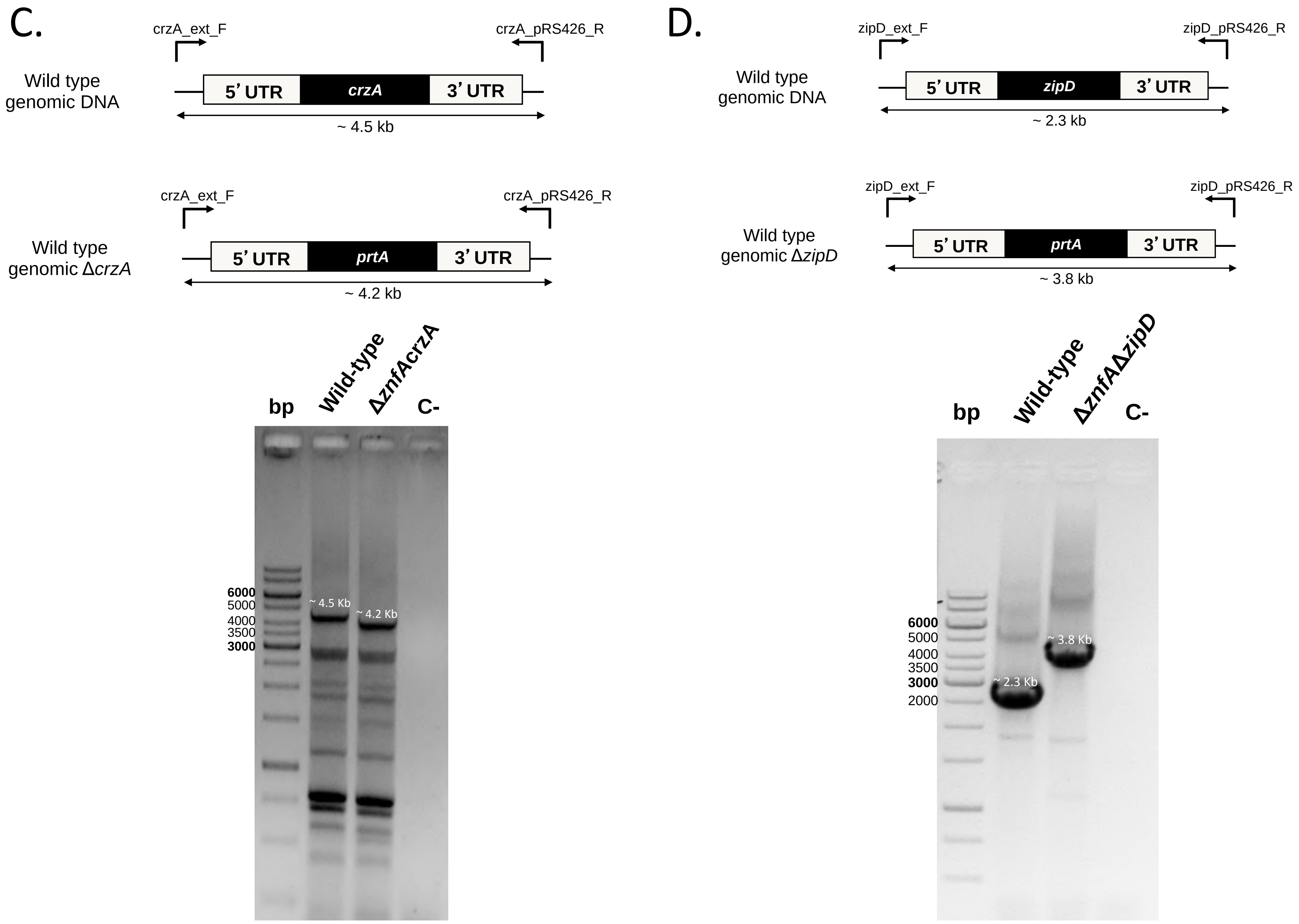
C.
crzA_ext_F
crzA_pRS426_R
Wild type genomic DNA
5' UTR
crzA
3' UTR
~ 4.5 kb
crzA_ext_F
crzA_pRS426_R
Wild type genomic ΔcrzA
5' UTR
prtA
3' UTR
~ 4.2 kb
bp
Wild-type
ΔznfAcrzA
C-
6000
5000
4000
3500
3000
~ 4.5 Kb
~ 4.2 Kb
D.
zipD_ext_F
zipD_pRS426_R
Wild type genomic DNA
5' UTR
zipD
3' UTR
~ 2.3 kb
zipD_ext_F
zipD_pRS426_R
Wild type genomic ΔzipD
5' UTR
prtA
3' UTR
~ 3.8 kb
bp
Wild-type
ΔznfAΔzipD
C-
6000
5000
4000
3500
3000
2000
~ 3.8 Kb
~ 2.3 Kb
