# Supplementary material for: Aspergillus Fumigatus ZnfA, a Novel Zinc Finger Transcription Factor Involved in Calcium Metabolism and Caspofungin Tolerance
Source: Front Fungal Biol. 2021 Aug 10;2:689900. doi: 10.3389/ffunb.2021.689900 (PMC10512341; doi:10.3389/ffunb.2021.689900)
Supplement: Supplementary Table 2 — MIC values and ranges (μg/ml) obtained by CLSI antifungal susceptibility testing method for the A. fumigatus strains included in this work. [file Table_2.DOCX]

**Supplementary Table S2.** MIC values and ranges (μg/ml) obtained by CLSI antifungal susceptibility testing method for the *A. fumigatus* strains included in this work.

|  | Amphotericin B | Voriconazole | Itraconazole | Posaconazole |
| --- | --- | --- | --- | --- |
| WT (CEA17) | 1-2 | 0.25-0.5 | 0.25-0.5 | 1 |
| *ΔznfA* | 1-2 | 0.25-0.5 | 0.25-0.5 | 0.5-1 |
| *ΔcrzA* | 1-2 | 0.25-0.5 | 0.25-0.5 | 0.5-1 |
| *ΔzipD* | 2 | 0.25-0.5 | 0.25-0.5 | 0.5-1 |
| *ΔznfAΔcrzA* | 2 | 0.25-0.5 | 0.25-0.5 | 0.5-1 |
| *ΔznfAΔzipD* | 2 | 0.25 | 0.25 | 1 |
| *ΔznfA::znfA*^+^ | 2 | 0.25-0.5 | 0.25-0.5 | 1 |
| *ΔcrzA::crzA*^+^ | 2 | 0.25-0.5 | 0.25-0.5 | 1 |
| *ΔzipD::zipD*^+^ | 2 | 0.25-0.5 | 0.25-0.5 | 0.5-1 |
