# Supplementary material for: Aspergillus Fumigatus ZnfA, a Novel Zinc Finger Transcription Factor Involved in Calcium Metabolism and Caspofungin Tolerance
Source: Front Fungal Biol. 2021 Aug 10;2:689900. doi: 10.3389/ffunb.2021.689900 (PMC10512341; doi:10.3389/ffunb.2021.689900)
Supplement: Supplementary Table 3 — List of primers used in this work. [file Table_3.DOCX]

**Supplemetary Table S3.** List of primers used in this work.

Strain constructions

| **Gene/**  **plasmid** | **Primer** | **Sequence (5'-3')** |
| --- | --- | --- |
| **pOB430/**  **pOB435** | OZG916_F | GGAGGTGGTAGCGGTGGT |
|  | OZG964_R | CTGTCTGAGAGGAGGCACTGA |
| **pPRTI** | prtA_F | GATCTGACAGACGGGCAATTG |
|  | prtA_R | CTATCATGGGGTGACGATGAGCCG |
| ***znfA*** | znfA_ext_F | CTTTACGCAACAGAACTTACC |
|  | znfA_ext_R | TCTCCGATTAGCAAAAGAAGC |
|  | znfA_pRS426_UTR5'_F | GTAACGCCAGGGTTTTCCCAGTCACGACGTTCATTTCTGGTGGATTTTC |
|  | znfA_linker_GFP/HA_ORF_R | ACCACCGCTACCACCTCCACACGGGCTCAGAACCTCAC |
|  | znfA_linker_ORF_F | ATCAGTGCCTCCTCTCAGACAGTCCGTTTGGTGAAGGAGCAA |
|  | znfA_pRS426_UTR3'_R | GCGGATAACAATTTCACACAGGAAACAGCGAAGCTGCCGGGTTGATTG |
|  | znfA_linker_ORF_prtA_F | CGGCTCATCGTCACCCCATGATAGTCCGTTTGGTGAAGGAGCAA |
| ***crzA*** | crzA_ext_F |  |
|  | crzA_pRS426_UTR5'_F | TGTCTATTCTGGGATCACGTTGATCTGACAGACGGGCAATTG |
|  | crzA _link_prtA_R | CAATTGCCCGTCTGTCAGATCAACGTGATCCCAGAATAGACA |
|  | crzA _link_prtA_F | CGGCTCATCGTCACCCCATGATAGTGAACCATCCGGTCAGATTCC |
|  | crzA _pRS426_UTR5'_R | GCGGATAACAATTTCACACAGGAAACAGCGCAGGCCTGATCAGCGTCGTCCAC |
| ***zipD*** | zipD_ext_F | CTTTATCTGGTTAGACATTTGTAC |
|  | zipD_pRS426_UTR5'_F | GTAACGCCAGGGTTTTCCCAGTCACGACGCATAATTTAATATGGTTCCAATGAAC |
|  | zipD_link_prtA_R | CAATTGCCCGTCTGTCAGATCCCCGGTACTTCCGCAATGGTGGCG |
|  | zipD_link_prtA_F | CGGCTCATCGTCACCCCATGATAGCGTGTGCGCCACCTTAGACCC |
|  | zipD_pRS426_UTR5'_R | GCGGATAACAATTTCACACAGGAAACAGCCTTTCAGAGCCAAACAAACACAGC |

RT-qPCR

| **Gene** | **Primer** | **Sequence (5'-3')** |
| --- | --- | --- |
| ***act1*** | act1_Afu6g04740_qPCR_F | CCTCCTGAGCGTAAATACTC |
|  | act1_Afu6g04740_qPCR_R | GAAGGACCGCTCTCGT |
| ***mirD*** | mirD_Afu3g03440_qPCR_F | GGTCGGTCGGGTACATGATC |
|  | mirD_Afu3g03440_qPCR_R | GTAGATCGTCTGCCAGTCCG |
| ***sidG*** | sidG_Afu3g03650_qPCR_F | TCGTGGGTTGGTGGGTATTG |
|  | sidG_Afu3g03650_qPCR_R | CTGGCGGTGGAGTATCGTAC |
| ***sidJ*** | sidJ_Afu3g03650_qPCR_F | ATGATCAGGGAGCAGGGTCT |
|  | sidJ_Afu3g03650_qPCR_R | TGACCAGAAAGTTCCGAGGC |
| ***sit1*** | sit1_Afu7g06060_qPCR_F | GGCATCTACCTCGCAACGTA |
|  | sit1_Afu7g06060_qPCR_R | GGATTGCGGATACAGAGCGA |
| ***zfpA*** | zfpA_Afu4g07090_qPCR_F | CCCGCGCAACTATTTCGAAG |
|  | zfpA_Afu4g07090_qPCR_R | CTTTTCATCCTCGTTGGCGC |
| ***znfA*** | znfA_Afu8g05010_qPCR_F | TGCGGATCATTTTCGACAGC |
|  | znfA_Afu8g05010_qPCR_R | ACGCGTTTTCCAAGACGTTG |
| ***fks1*** | fksA_Afu6g12400_FW | AAGCAATCGAAGCTCAGGAA |
|  | fksA_Afu6g12400_REV | ACCAATCCCATAGAGCGAAC |
| ***chiA1*** | Afu5g03760 sybr Fw | GTCCCTCGTTTCCACCTACA |
|  | Afu5g03760 sybr Rv | GATGGGATAGCACTGGAGGA |
